# The 2023 Guidelines for the management and treatment of glucocorticoid-induced osteoporosis

**DOI:** 10.1007/s00774-024-01502-w

**Published:** 2024-03-28

**Authors:** Yoshiya Tanaka, Satoshi Soen, Shintaro Hirata, Yosuke Okada, Saeko Fujiwara, Ikuko Tanaka, Yuriko Kitajima, Takuo Kubota, Kosuke Ebina, Yuichi Takashi, Reiko Inoue, Mika Yamauchi, Naoaki Okubo, Masanobu Ueno, Yasuhisa Ohata, Nobuaki Ito, Keiichi Ozono, Hisanori Nakayama, Masakazu Terauchi, Sakae Tanaka, Seiji Fukumoto

**Affiliations:** 1 The First Department of Internal Medicine, School of Medicine, University of Occupational and Environmental Health, Japan, 1-1 Iseigaoka, Yahata-Nishi, Kitakyushu, 807-8555 Japan; 2 Soen Orthopaedics, Osteoporosis and Rheumatology Clinic, Kobe, Japan; 3 Department of Clinical Immunology and Rheumatology, Hiroshima University Hospital, Hiroshima, Japan; 4 Clinical Research Center, Hospital of the University of Occupational and Environmental Health, Kyoto, Japan; 5 Department of Pharmacy, Yasuda Women’s University, Hiroshima, Japan; 6 Initiative for Rheumatology & Osteoporosis, Nagoya Rheumatology Clinic, Nagoya, Japan; 7 Department of Obstetrics and Gynecology, Nagasaki University Hospital, Nagasaki, Japan; 8 Department of Pediatrics, Osaka University Graduate School of Medicine, Osaka, Japan; 9 Department of Orthopaedic Surgery, Osaka University Graduate School of Medicine, Osaka, Japan; 10 Department of Musculoskeletal Regenerative Medicine, Osaka University Graduate School of Medicine, Osaka, Japan; 11 Department of Endocrinology and Diabetes Mellitus, Fukuoka University School of Medicine, Fukuoka, Japan; 12 Third Department of Medicine, Teikyo University Chiba Medical Center, Chiba, Japan; 13 Research Institute for Metabolic Bone Diseases, Eikokai Ono Hospital, Ono, Japan; 14 Division of Nephrology and Endocrinology, The University of Tokyo Hospital, Tokyo, Japan; 15 Osteoporosis Center, The University of Tokyo Hospital, Tokyo, Japan; 16 Department of Rheumatology, Soshigayaokura-Clinic, Setagaya, Tokyo, Japan; 17 Department of Women’s Health, Tokyo Medical and Dental University, Tokyo, Japan; 18 Department of Orthopaedic Surgery, Faculty of Medicine, The University of Tokyo, Tokyo, Japan; 19 Fujii Memorial Institute of Medical Sciences, Institute of Advance Medical Sciences, Tokushima University, Tokushima, Japan

**Keywords:** Glucocorticoid, Osteoporosis, Glucocorticoid-induced osteoporosis, Fracture, Treatment

## Abstract

**Introduction:**

Although synthetic glucocorticoids (GCs) are commonly used to treat autoimmune and other diseases, GC induced osteoporosis (GIOP) which accounts for 25% of the adverse reactions, causes fractures in 30–50% of patients, and markedly decreases their quality of life. In 2014, the Japanese Society for Bone and Mineral Research (JSBMR) published the revised guidelines for the management and treatment of steroid-induced osteoporosis, providing the treatment criteria based on scores of risk factors, including previous fractures, age, GC doses, and bone mineral density, for patients aged ≥18 years who are receiving GC therapy or scheduled to receive GC therapy for ≥3 months.

**Materials and methods:**

The Committee on the revision of the guidelines for the management and treatment of GIOP of the JSBMR prepared 17 clinical questions (CQs) according to the GRADE approach and revised the guidelines for the management and treatment of GIOP through systematic reviews and consensus conferences using the Delphi method.

**Results:**

Bisphosphonates (oral and injectable formulations), anti-RANKL antibody teriparatide, eldecalcitol, or selective estrogen receptor modulators are recommended for patients who has received or scheduled for GC therapy with risk factor scores of ≥3. It is recommended that osteoporosis medication is started concomitantly with the GC therapy for the prevention of fragility fractures in elderly patients.

**Conclusion:**

The 2023 guidelines for the management and treatment of GIOP was developed through systematic reviews and consensus conferences using the Delphi method.

**Supplementary Information:**

The online version contains supplementary material available at 10.1007/s00774-024-01502-w.

## Introduction

Glucocorticoids (GCs) are secreted from the adrenal cortex upon stimulation by adrenocorticotrophic hormone from the hypothalamus-anterior pituitary system. Endogenous GCs bind to the GC receptor (GR) and translocate into the nucleus to regulate the physiological metabolism of glucose, lipid, and bone, among others, through transcription, thereby maintaining body homeostasis. Synthetic GCs are widely used to treat various diseases in different fields, including autoimmune rheumatic diseases, respiratory, renal, neurological, and allergic diseases, and graft rejection, through pharmacological actions, such as potent anti-inflammatory and immunosuppressive actions. In treating these diseases, synthetic GCs exert pharmacological actions by regulating the transcription of proinflammatory mediators through GR. However, synthetic GCs also bind to GR to cause abnormal metabolism of glucose, lipid, bone, and blood vessels, among others [1–3].

Abnormal bone metabolism induced by GCs is called GC-induced osteoporosis (GIOP). It is common, accounts for 25% of adverse drug reactions, and affects 0.7–1.2% of adults [4, 5]. GCs inhibit mesenchymal stem cell differentiation into osteoblasts and induce osteoblast and osteocyte apoptosis, inhibiting bone matrix production and then reducing bone mass and quality [1–3]. Simultaneously, GCs, directly and indirectly, stimulate osteoclast maturation and activation. Consequently, GCs rapidly reduce bone quality as well as quality and osteoporotic changes rapidly progress at 3–6 months after administration. GCs also cause fragility fractures in 30–50% of the long-term administrated patients [Suppl. Ref. 17]. No safety margin exists for dose-dependent bone loss even at low GC dosages; osteoporotic effects are inevitable.

GIOP is a frequently occurring adverse drug reaction to prescribed drugs. Fragility fractures associated with osteoporotic changes markedly decrease quality of life (QOL). Therefore, the Japanese Society for Bone and Mineral Research (JSBMR) developed the guidelines for the management and treatment of steroid-induced osteoporosis in 2004 [6]. Furthermore, the society revised the guidelines in 2014, recommending general guidance for patients aged ≥ 18 years who has already received GC therapy or scheduled to receive GC therapy for ≥ 3 months and treatment interventions when their scores of risk factors, including previous fractures, age, GC doses, and bone mineral density are ≥ 3 points, based on Japanese evidence [7].

The subsequent vigorous development of anti-osteoporotic drugs and evaluation of the drugs for GIOP have accumulated enormous evidence. Thus, the JSBMR guideline revision committee prepared 17 clinical questions (CQs) according to the Grading of Recommendations, Assessment, Development, and Evaluations (GRADE) approach and performed systematic reviews to develop the best recommendations for management and treatment based on scientific evidence and expert views. In addition, the committee held consensus conferences using the Delphi method to prepare recommendations for each CQ, for which the recommendation grades and consensus levels were shown.

## Materials and methods

### Guideline development committee and policies

The JSBMR guideline development committee took the leading role in the development of guidelines for the management and treatment of GIOP. Additionally, the steering committee on clinical practice guidelines, chaired by the president of the society, clarified the objectives, system determination, and internal evaluations, among others. Furthermore, a systematic review team was formed mainly by the society.

The Minds Handbook for Clinical Practice Guideline Development was used to develop the 2023 guidelines for the management and treatment of GIOP, which aimed to present optimal recommendations for supporting decision making of patients and healthcare professionals in consideration of balancing risks and benefits and to provide evidence-based medicine for clinically important medical practices [8]. The scope of the guidelines was determined to cover overall aspects of GIOP, such as understanding the epidemiology, clinical conditions, disease pathology, management, and treatment. While presenting each item in the CQ format, the committee aimed to develop efficient guidelines that general clinicians could quickly understand and implement in their clinical practice. The committee also aimed to spread the guidelines afterward widely. The guidelines were developed to be comprehensible by general physicians who were not osteoporosis specialists and by professionals in various fields. The committee also aimed to develop guidelines to provide the materials for making decisions on standardized medical care so that all patients with GIOP could equally receive appropriate treatment.

The scope was set as follows. 1) To discuss GIOP drug therapy initiation criteria. 2) To propose drugs effective for increasing bone mineral density and preventing fractures in patients who scheduled to receive GC therapy for prevention (including those received GCs for ≤ 3 months), according to the GRADE approach. 3) To propose drugs effective for increasing bone mineral density and preventing fractures in patients who has already received GCs for ≥ 3 months for treatment, according to the GRADE approach. The outcomes were fragility fractures, bone mineral density/bone quality, death, continuation rate, hypercalcemia/hypocalcemia, hypercalciuria, activities of daily living, QOL, anti-resorptive agent-related osteonecrosis of the jaw, atypical fractures, the number of operations, fertility, tolerability, labor productivity, job separation rate, and impact on the primary diseases.

### Guideline development process

The committee prepared 17 CQs regarding the overall aspects of GIOP and performed systematic reviews. Each CQ was formulated in the PICO (patients, interventions, comparison, and outcomes) format. Keywords were selected for the PICO elements and used as search words to formulate a search equation. Infront Medical Publications was commissioned to prepare a literature search list using the PubMed and Scopus databases on articles published in and after 2000.

The systematic review teams used the literature search list to perform the primary screening, which a team of two members independently performed for the abstract of each listed article, and the secondary screening, which another team of two members independently performed to examine the full texts of the screened articles. The predefined bias risks were evaluated for the selected articles to determine the evidence levels. The guideline development committee received a summary report with evidence levels, recommendation grades, and draft recommendations. Based on the third version of the Minds Handbook for Clinical Practice Guideline Development, the committee evaluated evidence levels on a 4-point scale of A (strong), B (moderate), C (weak), and D (very weak). When meta-analysis was impossible, recommendations were developed based on narrative reviews.

The guideline development committee evaluated the recommendation grades by using the recommendation classification of the Minds Handbook for Clinical Practice Guideline Development. Recommendation grade 1: Implementation is strongly recommended. Recommendation grade 2: Implementation is weakly recommended (proposed). Recommendation grade 3: Non-implementation is weakly recommended (proposed). Recommendation grade 4: Non-implementation is strongly recommended. Clinical studies and academic articles with high evidence levels have better recommendation grades for test methods and treatment options. The evidence levels were compared to the recommendation classification.

Meanwhile, as described in the Minds Handbook for Clinical Practice Guideline Development, the strength of evidence is not directly equivalent to the strength of recommendation. Holding consensus conferences to determine recommendations and recommendation grades through unbiased decision-making approaches is also preferable. To compensate for low evidence levels, the guideline development committee voted on a 9-point scale using the Delphi method, and the criteria for adoption were set at ≥ 8.0 points. Recommendations were revised until they were adopted. Additionally, this committee prepared the literature by summarizing the literature extraction process, background, explanation, and scientific evidence regarding the recommendations.

Furthermore, the steering committee on clinical practice guidelines repeatedly discussed, polished, and revised the recommendations. Public comments were widely sought through the JSMBR and reflected in the contents of this article.

## Results

The 17 CQs prepared regarding the overall aspects of GIOP were systematically reviewed: CQs 1 to 6 for epidemiology, clinical features, pathology, and criteria for treatment initiation; CQs 7 to 13 on therapeutic drug efficacy in GIOP for increasing bone mineral density and preventing fractures, which were the most important outcomes in both patients scheduled to receive GC therapy and patients who have already received GC therapy for ≥ 3 months; and CQs 14 to 16 for management and treatment of children, elderly patients, and women of childbearing age and CQ 17 for surgical treatments of fragility fractures. A literature search yielded 1,413 and 1,426 articles published in or after 2000 that contained the terms “glucocorticoid-induced” or “steroid-induced,” “osteoporosis” or “fracture,” and “GIOP” using PubMed and Scopus, respectively. After articles that were inapplicable to the CQs were excluded by the primary screening, the secondary screening was performed to assess eligibility of literatures. Subsequently, a systematic literature review of all 17 CQs was performed, using randomized clinical trials (RCTs) as the target of qualitative as well as quantitative evaluation, and applicable RCTs and/or meta-analysis were found for CQs 7–13. The search formula, strategy and flow chart were shown in each CQs 7–13 and Supplementary Figures (Suppl Figs. 1, 2, 3, 4, 5, 6, 7). In contrast, because applicable RCTs were not found for CQs 1–6 and 14–17, we decided to conduct a narrative reviews of cohort studies, large-scale cross-sectional studies, epidemiological studies, and patient statistics using mainly relevant literature as expert opinion (Table 1). The following section presents the CQs, recommendations, evidence levels, recommendation grades, consensus levels, and brief explanations.

**Table 1:** Clinical questions (CQs) for the management and treatment of glucocorticoid-induced osteoporosis

CQ#	CQ	Recommendation	Evidence level	Recommendation grade	Consensus level
1	How many patients with GIOP are there? What are their QOL and outcomes?	Patients with GIOP account for 0.7–1.2% of adults in the world. Since fractures occur in 30–50% of the patients and markedly impair their QOL, appropriate management and treatment of this disease are recommended	D	1	9.0
2	What are the risk factors for GIOP?	The risk factors of GIOP include advanced age, GC doses, low bone mineral density of the lumbar spine, presence of previous fractures, and no receipt of bisphosphonate therapy. It is recommended to implement appropriate management and treatment of GIOP without delay while attention is paid to these risk factors	D	1	9.0
3	How is the development of GIOP associated with the GC doses?	Since the development of GIOP depends on the dose and duration of GC therapy, it is recommended to initiate the administration of GCs at the lowest dose possible and to immediately reduce the GC doses according to the pathological condition of the primary disease	D	1	8.9
4	What are the symptoms, examinations, and imaging findings useful for diagnosing GIOP?	Although GIOP has no specific symptoms, vertebral fractures are useful for the diagnosis. It is recommended to evaluate whether there are vertebral fractures soon after initiating GC therapy	D	1	8.6
		During GC therapy, it is recommended to measure bone mineral density by dual-energy X-ray absorptiometry regularly	D	1	8.4
5	What is the guidance on lifestyle and nutrition for patients with GIOP?	It is recommended to prioritize the treatment of the primary disease, which is the reason for the administration of GCs, and to guide lifestyle, including nutrition, according to the disease characteristics of the primary disease	D	1	9.0
		It is recommended to guide lifestyle and nutrition according to the Japanese guidelines for preventing and treating osteoporosis	D	1	9.0
6	What are the criteria for initiating drug therapy for GIOP?	It is recommended to use the cut-off scores described in the “Guidelines on the management and treatment of GIOP of the JSBMR: 2014 update" as the criteria for initiating drug therapy for GIOP	C	1	9.0
7	Are active vitamin D preparations useful for treating GIOP?	Active vitamin D preparations, such as eldecalcitol, are recommended because they effectively increase the bone mineral density of the lumbar spine and prevent non-vertebral fractures in patients scheduled to receive GC therapy and patients receiving GC therapy	B	1	8.0
8	Are bisphosphonates useful for the treatment of GIOP?	Bisphosphonates are recommended for patients scheduled to receive GC therapy or patients receiving GC therapy because there is evidence for the effects of bisphosphonates in increasing the bone mineral density of the lumbar spine and femur and preventing vertebral and non-vertebral fractures	A	1	9.0
9	Are selective estrogen receptor modulators useful for the treatment of GIOP?	Although there is no evidence that selective estrogen receptor modulators (SERMs) are effective for preventing vertebral or non-vertebral fractures in postmenopausal women at risk of GIOP, they are effective for increasing the bone mineral density of the lumbar spine and femur. Thus, the use of SERMs is proposed	C	2	8.1
10	Are parathyroid hormone 1 receptor agonists useful in treating GIOP?	Parathyroid hormone 1 (PTH1) receptor agonists are recommended for patients at high risk of fractures because these agonists can be expected to be effective in increasing the bone mineral density of the lumbar spine and preventing vertebral fractures in patients scheduled to receive GC therapy and patients receiving GC therapy	B	1	8.8
11	Is an anti-RANKL antibody useful for the treatment of GIOP?	An anti-RANKL antibody should be administered to patients scheduled for or receiving GC therapy to increase lumbar spine and femur bone mineral density and prevent vertebral fractures	B	1	8.9
12	Is an anti-sclerostin antibody useful for the treatment of GIOP?	(future study issue)			
13	Are the above-described drugs different in terms of usefulness?	Recombinant teriparatide and an anti-RANKL antibody are more effective than bisphosphonates for preventing vertebral fractures. The use of the former drugs is recommended. Recombinant teriparatide is recommended for patients at high risk of fractures	B	1	8.0
14	How is GIOP prevented and treated in children?	Bisphosphonates are proposed for preventing and treating GIOP in children	D	2	8.0
15	How is GIOP prevented and treated in elderly patients?	In elderly patients, intervention with anti-osteoporotic drugs in combination with GC therapy is recommended to prevent and treat fractures	D	1	8.1
16	How is GIOP prevented and treated in women of childbearing age?	It is recommended to guide lifestyle and nutrition to women of childbearing age who are scheduled to receive GC therapy and those who are receiving GC therapy, according to Guidelines on the management and treatment of GIOP of the JSBMR: 2014 update	D	1	8.3
		It is recommended that bisphosphonates, an anti-RANKL antibody, and PTH1 receptor agonists should not be administered to pregnant or breastfeeding women for the treatment of GIOP	D	4	8.3
17	What is the surgical treatment of fragility fractures associated with GIOP?	For surgical treatment of fragility fractures attributable to GIOP, treatment strategies according to the treatment of primary osteoporosis are recommended	D	1	8.1

## CQ 1: How many patients with GIOP are there? What are their QOL and outcomes?

[Recommendation] Patients with GIOP account for 0.7–1.2% of adults in the world. Since fractures occur in 30–50% of the patients and markedly impair their QOL, appropriate management and treatment of this disease are recommended.

### Evidence level: D; recommendation grade: 1; consensus level: 9.0

By the primary and secondary screenings in the process of systemic literature review, RCTs and meta-analysis were not found for the CQ1. A narrative reviews of cohort studies, large-scale cross-sectional studies, epidemiological studies and patient statistics was, therefore, conducted using 7 extracted relevant literatures with newly added review papers to prepare the recommendations.

Based on articles published in countries globally, approximately 0.7–1.2% of adults develop osteoporosis because of long-term GC therapy [Suppl. Ref. 1–21]. There is scientific evidence that the number of patients increases with age. Fractures occur in 30–50% of these patients and markedly impair their QOL. While the risk of vertebral fractures is increased by 55% even at a prednisolone-equivalent dose of < 2.5 mg, the relative risk of vertebral fractures is increased by 14 folds in patients who are treated at a dose of ≥ 15 mg/day and have a cumulative exposure level of ≥ 5 g. This disease is an iatrogenic adverse drug reaction that occurs frequently. According to the 2014 revised guidelines with strong scientific evidence, the importance of management and treatment should be reacknowledged [7].

## CQ 2: What are the risk factors for GIOP?

[Recommendation] The risk factors of GIOP include advanced age, GC doses, low bone mineral density of the lumbar spine, presence of previous fractures, and no receipt of bisphosphonate therapy. It is recommended to implement appropriate management and treatment of GIOP without delay while attention is paid to these risk factors.

### Evidence level: D; recommendation grade: 1; consensus level: 9.0

The risk factors of GIOP detected in the current revision were identical to those extracted by the analysis performed on three cohort studies on Japanese patients to prepare the 2014 revised guidelines [Suppl. Ref. 22–32]. This further emphasizes the importance of management and treatment of GIOP according to the 2014 revised guidelines [7].

## CQ 3: How is the development of GIOP associated with the GC doses?

[Recommendation] Since the development of GIOP depends on the dose and duration of GC therapy, it is recommended to initiate the administration of GCs at the lowest dose possible and to immediately reduce the GC doses according to the pathological condition of the primary disease.

### Evidence level: D; recommendation grade: 1; consensus level: 8.9

The 6 articles extracted by systemic literature review were used as the basis for the narrative review. GCs cause GIOP as well as abnormal metabolism such as glucose and lipid. Simultaneously, GCs are a risk factor for opportunistic infections, cardiovascular disorders, and cerebrovascular disorders and many. Thus, it is emphasized that indication of GCs should be decided carefully and GCs should be administered at the minimum necessary dose for the shortest duration after considering whether the benefits of GC therapy outweigh the risks [Suppl. Ref. 33–38].

## CQ 4: What are the symptoms, examinations, and imaging findings useful for diagnosing GIOP?

[Recommendation] (1) Although GIOP has no specific symptoms, vertebral fractures are useful for the diagnosis. It is recommended to evaluate whether there are vertebral fractures soon after initiating GC therapy.

### Evidence level: D; recommendation grade: 1; consensus level: 8.6

(2) During GC therapy, it is recommended to measure bone mineral density by dual-energy X-ray absorptiometry regularly.

### Evidence level: D; recommendation grade: 1; consensus level: 8.4

Both recommendations are based on analytical epidemiological studies. Hence, the evidence level was D. However, vertebral fractures are the most frequently occurring osteoporosis-related fractures and fragility-fractures often cause severe pain. Also, asymptomatic vertebral fractures account for two-thirds of all osteoporosis-related fractures. Moreover, a pre-existing fracture, with a score of 7 points, carries the greatest risk of a second fracture among the risk factors for fracture. Accordingly, the recommendation grade was 1 because advantages of the recommended measurements outweigh the risk and burdens [Suppl. Ref. 39–65] and evaluation of vertebral fracture is mandatory in patients receiving glucocorticoids. Basically, GIOP is diagnosed using the "treatment intervention criteria for management and prevention" instead of the "diagnostic criteria." It is ideal for pre-fracture treatment.

## CQ 5: What is the guidance on lifestyle and nutrition for patients with GIOP?

[Recommendation] (1) It is recommended to prioritize the treatment of the primary disease, which is the reason for the administration of GCs, and to guide lifestyle, including nutrition, according to the disease characteristics of the primary disease.

### Evidence level: D; recommendation grade: 1; consensus level: 9.0

(2) It is recommended to guide lifestyle and nutrition according to the Japanese guidelines for preventing and treating osteoporosis.

### Evidence level: D; recommendation grade: 1; consensus level: 9.0

Primary disease activity, organ dysfunction (e.g., the kidney), menopause, low body mass index (BMI), advanced age, recumbency, and dysfunction enhance osteoporotic changes. GC therapy should improve primary disease activity. However, case-by-case evaluation is important because of the additional impacts of GCs on calcium and bone metabolism. Exercise and nutritional interventions, including calcium and vitamin D supplementation, are necessary to maximize therapeutic agent benefits [Suppl. Ref. 66–95].

## CQ 6: What are the criteria for initiating drug therapy for GIOP?

[Recommendation] It is recommended to use the cut-off scores described in the “Guidelines on the management and treatment of GIOP of the JSBMR: 2014 update" as the criteria for initiating drug therapy for GIOP.

### Evidence level: C; recommendation grade: 1; consensus level: 9.0

The 6 articles extracted by systemic literature review were used as the basis for the narrative review. No clear intervention threshold based on FRAX® for initiating drug therapy has been determined in Japan. However, when the 2014 revised guidelines were prepared, risk factors for fractures were extracted by analyzing three cohort studies on Japanese patients and weighted based on parameter estimates to develop a scoring system and to determine cut-off values [7]. Thus, using the cut-off scores described in the 2014 revised guidelines as the criteria for initiating drug therapy for GIOP is strongly recommended [Suppl. Ref. 96–110].

## CQ 7: Are active vitamin D preparations useful for treating GIOP?

[Recommendation] Active vitamin D preparations, such as eldecalcitol, are recommended because they effectively increase the bone mineral density of the lumbar spine and prevent non-vertebral fractures in patients scheduled to receive GC therapy and patients receiving GC therapy.

### Evidence level: B; recommendation grade: 1; consensus level: 8.0

Of the extracted literature, 197 articles were selected for primary screening and 25 articles were selected for secondary screening. Finally, 9 papers were selected as literature eligible for recommendation and network meta-analysis, meta-analysis, and results of RCTs and comparative clinical trials (CCTs) were integrated and described as a systematic review (Suppl Fig. 1).

One RCT demonstrated that alfacalcidol increased femoral neck bone mineral density better than natural vitamin D. Eldecalcitol increased lumbar spine, proximal femur, and femoral neck bone mineral density than alfacalcidol in multiple RCTs and meta-analysis. Thus, the recommendation grade was 1 for active vitamin D preparations such as eldecalcitol in patients receiving and those scheduled to receive GC therapy [Suppl. Ref. 111–127].

## CQ 8: Are bisphosphonates useful for the treatment of GIOP?

[Recommendation] Bisphosphonates are recommended for patients scheduled to receive GC therapy or patients receiving GC therapy because there is evidence for the effects of bisphosphonates in increasing the bone mineral density of the lumbar spine and femur and preventing vertebral and non-vertebral fractures.

### Evidence level: A; recommendation grade: 1; consensus level: 9.0

Of the extracted literature, 372 articles were selected for primary screening and 149 articles were selected for secondary screening. Finally, 7 articles were selected for the recommendation, including 5 RCTs, were integrated and described as a systematic review (Suppl Fig. 2).

Alendronate and risedronate were recommended at grade A in the 2014 revised guidelines [7], but there was no new negative evidence; therefore, the current recommendation grade is 1. The recommendation grade was high because an RCT involving young participants demonstrated that risedronate increased lumbar spine bone mineral density better than alfacalcidol. A Japanese RCT demonstrated that a combination of minodronate and alfacalcidol significantly increased lumbar spine and proximal femur bone mineral density more than alfacalcidol alone. The recommendation grade for this combination was 2. Ibandronate was superior to placebo in multiple RCTs, and its intravenous formulation temporarily prevented vertebral fractures. The recommendation grade for ibandronate was 1. Multiple RCTs and meta-analyses demonstrated that zoledronate significantly increased vertebral body bone mineral density than placebo or risedronate. The recommendation grade for zoledronate was 1 [Suppl. Ref. 128–135].

## CQ 9: Are selective estrogen receptor modulators useful for the treatment of GIOP?

[Recommendation] Although there is no evidence that selective estrogen receptor modulators (SERMs) are effective for preventing vertebral or non-vertebral fractures in postmenopausal women at risk of GIOP, they are effective for increasing the bone mineral density of the lumbar spine and femur. Thus, the use of SERMs is proposed.

### Evidence level: C; recommendation grade: 2; consensus level: 8.1

Of the extracted literature, 38 articles were selected for primary screening and 12 articles were selected for secondary screening. Finally, 8 papers were selected as literature eligible for recommendation and network meta-analysis, meta-analysis, and results of RCTs and comparative clinical trials (CCTs) were integrated and described as a systematic review (Suppl Fig. 3).

Regarding the use of SERMs for treating GIOP, two articles on meta-analyses showed that SERMs effectively increased the bone mineral density of the lumbar spine and femur, whereas RCTs provided no clear evidence for the preventive effect on vertebral or non-vertebral fractures. Thus, the evidence level was determined to be C, and the recommendation grade was determined to be 2 [Suppl. Ref. 136–143].

## CQ 10: Are parathyroid hormone 1 receptor agonists useful in treating GIOP?

[Recommendation] Parathyroid hormone (PTH) 1 receptor agonists are recommended for patients at high risk of fractures because these agonists can be expected to be effective in increasing the bone mineral density of the lumbar spine and preventing vertebral fractures in patients scheduled to receive GC therapy and patients receiving GC therapy.

### Evidence level: B; recommendation grade: 1; consensus level: 8.8

Of the extracted literature, 229 articles were selected for primary screening and 24 articles were selected for secondary screening. Finally, 11 articles were selected for the recommendation, including 7 RCTs, 2 network meta-analysis, 1 meta-analysis, and 1 cohort, were integrated and described as a systematic review (Suppl Fig. 4).

A recombinant teriparatide has been shown to increase bone mineral density in lumber spine and proximal femurs significantly more than alendronate and to prevent vertebral fractures in meta-analyses and network meta-analyses that examined both primary and secondary prevention. Based on multiple network meta-analyses comparing the usefulness of drugs in patients with GIOP, a recombinant teriparatide has been shown to be the most effective drug for preventing vertebral fractures. Teriparatide acetate has been demonstrated to significantly increase the bone mineral density of the lumbar spine in both primary and secondary prevention of GIOP. Considering that PTH1 receptor agonists are indicated for treating patients with primary osteoporosis who have high risk of fracture, and high prices of these drugs, the PTH1 receptor agonists are also recommended for patients with GIOP who have similarly high risk of fragility fractures. Thus, the evidence level was B, and the recommendation grade was 1 [Suppl. Ref.144–154]. The usefulness of abaloparatide for the treatment of GIOP cannot be determined because of the lack of studies.

## CQ 11: Is an anti-RANKL antibody useful for the treatment of GIOP?

[Recommendation] An anti-RANKL antibody should be administered to patients scheduled for or receiving GC therapy to increase lumbar spine and femur bone mineral density and prevent vertebral fractures.

### Evidence level: B; recommendation grade: 1; consensus level: 8.9

Of the extracted literature, 119 articles were selected for primary screening and 65 articles were selected for secondary screening. Finally, 14 articles were selected for the recommendation, including 4 RCTs on a bisphosphonate, 2 network meta-analysis, were integrated and described as a systematic review (Suppl Fig. 5).

Denosumab, an anti-RANKL antibody, showed significant differences in meta-analyses and RCTs on a bisphosphonate, and the effects of the antibody for preventing vertebral fractures and increasing the bone mineral density of the femur seemed to outweigh the safety issues, such as atypical femur fractures and osteonecrosis of the jaw. Therefore, the recommendation grade was 1. However, the evidence level was B because of the small number of available studies, the small scale of the studies, and the use of bisphosphonates as a control. In Europe and the United States, the drug price of denosumab is much more expensive than in Japan, which is disadvantageous for medical economics there [Suppl. Ref. 155–159]. In Japan, where the drug price of denosumab is almost the same as that of bisphosphonates and is cheaper than PTH1 receptor agonists, denosumab can be strongly recommended.

## CQ 12: Is an anti-sclerostin antibody useful for the treatment of GIOP?

[Recommendation] No studies on the efficacy of an anti-sclerostin antibody for treating GIOP have yet been published. Thus, no clear recommendations can be made. This is a future study research [Suppl. Ref. 160–163].

Because no clinical studies using an anti-sclerostin antibody have been conducted on the prevention and treatment of GIOP, the guideline revision committee could not make clear recommendations (Suppl Fig. 6). This is a future study issue.

## CQ 13: Are the above-described drugs different in terms of usefulness?

[Recommendation] Recombinant teriparatide and an anti-RANKL antibody are more effective than bisphosphonates for preventing vertebral fractures. The use of the former drugs is recommended. Recombinant teriparatide is recommended for patients at high risk of fractures.

### Evidence level: B; recommendation grade: 1; consensus level: 8.0

Of the extracted literature, 302 articles were selected for primary screening and 57 articles were selected for secondary screening. Finally, 4 meta-analyses were selected for the recommendation, were integrated and described as a systematic review (Suppl Fig. 7).

Based on multiple network meta-analyses comparing the usefulness of drugs in patients with GIOP, teriparatide and denosumab were more effective than bisphosphonates for preventing vertebral fractures. A recombinant teriparatide was efficacious, particularly in patients at high risk of fractures. However, studies on non-vertebral fractures have shown no risk reduction effect because of insufficient sample size and other factors, and few studies have examined the adverse events and tolerability [Suppl. Ref. 164–167].

## CQ 14: How is GIOP prevented and treated in children?

[Recommendation] Bisphosphonates are proposed for preventing and treating GIOP in children.

### Evidence level: D; recommendation grade: 2; consensus level: 8.0

Of the extracted literatures, 6 articles were selected as the basis for the narrative review. Despite limited evidence for GIOP in children, small-scale RCTs have been conducted. The recommendation grade was 2. The calcium requirement is relatively high in the growth phase, and GCs significantly impact bone metabolism. However, bone formation is active, and there have also been reports of cured cases of vertebral fractures. Thus, children cannot be treated in the same manner as adults. Growth disorders due to GCs, in addition to GIOP, is also a significant problem in autoimmune diseases (e.g., juvenile idiopathic arthritis), connective tissue diseases, nephrotic syndrome, hematologic malignancy, bronchial asthma, inflammatory bowel disease, and other diseases for which GCs are administered at pharmacological doses. The treatment strategies for growth disorders due to GC therapy are reducing GC doses and discontinuing GC therapy (switching to other drug products) [Suppl. Ref. 168–175].

## CQ 15: How is GIOP prevented and treated in elderly patients?

[Recommendation] In elderly patients, intervention with anti-osteoporotic drugs in combination with GC therapy is recommended to prevent fractures.

### Evidence level: D; recommendation grade: 1; consensus level: 8.1

Evidence was limited for treating and managing GIOP in elderly patients aged ≥ 65 years. Based on Japanese cohort studies, the hazard ratio of fractures in patients aged ≥ 65 years is approximately twice higher than that in patients < 50 years used as a reference. In elderly patients, because osteoporotic changes are promoted by factors such as organ dysfunction (e.g., the kidney), low BMI, advanced age, recumbency, immobility, and menopause, it is clear that GC therapy further increases the risk of fractures. Thus, intervention with anti-osteoporotic drugs was strongly recommended for older patients who would receive GC therapy for ≥ 3 months after consideration of the background and characteristics of individual older patients. It is clear that the most important thing is to avoid the use of GCs in the elderly patients. [Suppl. Ref. 176–188].

## CQ 16: How is GIOP prevented and treated in women of childbearing age?

[Recommendation] (1) It is recommended to guide lifestyle and nutrition to women of childbearing age who are scheduled to receive GC therapy and those who are receiving GC therapy, according to Guidelines on the management and treatment of GIOP of the JSBMR: 2014 update.

### Evidence level: D; recommendation grade: 1; consensus level: 8.3

(2) It is recommended that bisphosphonates, an anti-RANKL antibody, and PTH1 receptor agonists should not be administered to pregnant or breastfeeding women for the treatment of GIOP.

### Evidence level: D; recommendation grade: 4; consensus level: 8.3

There are no published analytical epidemiological studies or randomized/non-RCTs exclusively targeting women of childbearing age with GIOP, and the evidence levels of all available studies are limited. Of the extracted literatures, 8 articles were selected as the basis for preparing the recommendations. The CQ was prepared based on a comprehensive narrative review of studies, including a subgroup of premenopausal women, reviews, and guidelines. Maintenance of a healthy lifestyle for prevention is recommended in grade 1. In principle, it is recommended that no drug therapy should be administered to pregnant or breastfeeding women. The recommendation grade for this was 4. When it is necessary to administer drug therapy to fertile women at moderate or high risk of fractures, oral bisphosphonates and teriparatide are used as the first-line and second-line drugs, respectively [Suppl. Ref. 189–193].

## CQ 17: What is the surgical treatment of fragility fractures associated with GIOP?

[Recommendation] For surgical treatment of fragility fractures attributable to GIOP, treatment strategies according to the treatment of primary osteoporosis are recommended.

### Evidence level: D; recommendation grade: 1; consensus level: 8.1

Typical osteoporotic fragility fractures are vertebral fractures and hip fractures. Other major osteoporotic fractures include distal radius fractures and proximal humerus fractures. Patients with GIOP are at high risk of fractures and may need surgery. There is no clear evidence on surgical treatment frequency and outcomes, specifically in patients with GIOP; however, many guidelines recommend that appropriate surgical treatment of proximal femur fractures and others improve the function. Thus, the selection of treatment strategies according to these guidelines is important [Suppl. Ref. 194–196].

## Conclusion

**Fig. 1:**
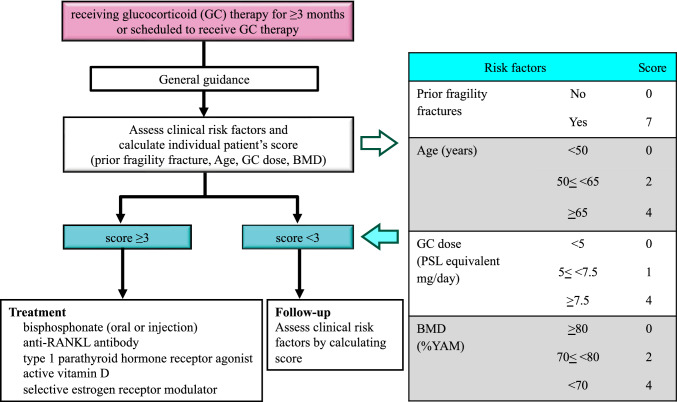
The 2023 Guidelines for the Management and Treatment of Glucocorticoid-induced Osteoporosis published by the Japanese Society for Bone and Mineral Research. BMD bone mineral density, GC glucocorticoid, PSL prednisolone, YAM young adult means

The Committee on the revision of the guidelines for the management and treatment of GIOP of the JSBMR prepared 17 CQs according to the GRADE approach and developed the "2023 guidelines for the management and treatment of GIOP" through systematic reviews and consensus conferences using the Delphi method. These guidelines recommended the use of bisphosphonates (oral and injectable formulations), an anti-RANKL antibody, teriparatide, eldecalcitol, or SERMs, in addition to general guidance for patients scheduled to receive GC therapy and patients receiving GC therapy, if their risk factor scores are calculated to be ≥ 3 points by the algorithm described in the 2014 revised guidelines (Fig. 1). Furthermore, osteoporosis medication should be done concomitantly with the GC therapy for the prevention of fragility fracture in elderly patients.

## Supplementary Information

Below is the link to the electronic supplementary material.

Supplementary file1 (PPTX 594 KB)

Supplementary file2 (DOCX 41 KB)

## Data Availability

The manuscript does not contain data to be shared.
