# Supplementary material for: The 2023 Guidelines for the management and treatment of glucocorticoid-induced osteoporosis
Source: J Bone Miner Metab. 2024 Mar 28;42(2):143–54. doi: 10.1007/s00774-024-01502-w (PMC10982086; doi:10.1007/s00774-024-01502-w)
Supplement: Supplementary file 1 — Supplementary file1 (PPTX 594 KB) [file 774_2024_1502_MOESM1_ESM.pptx]

## Slide 1
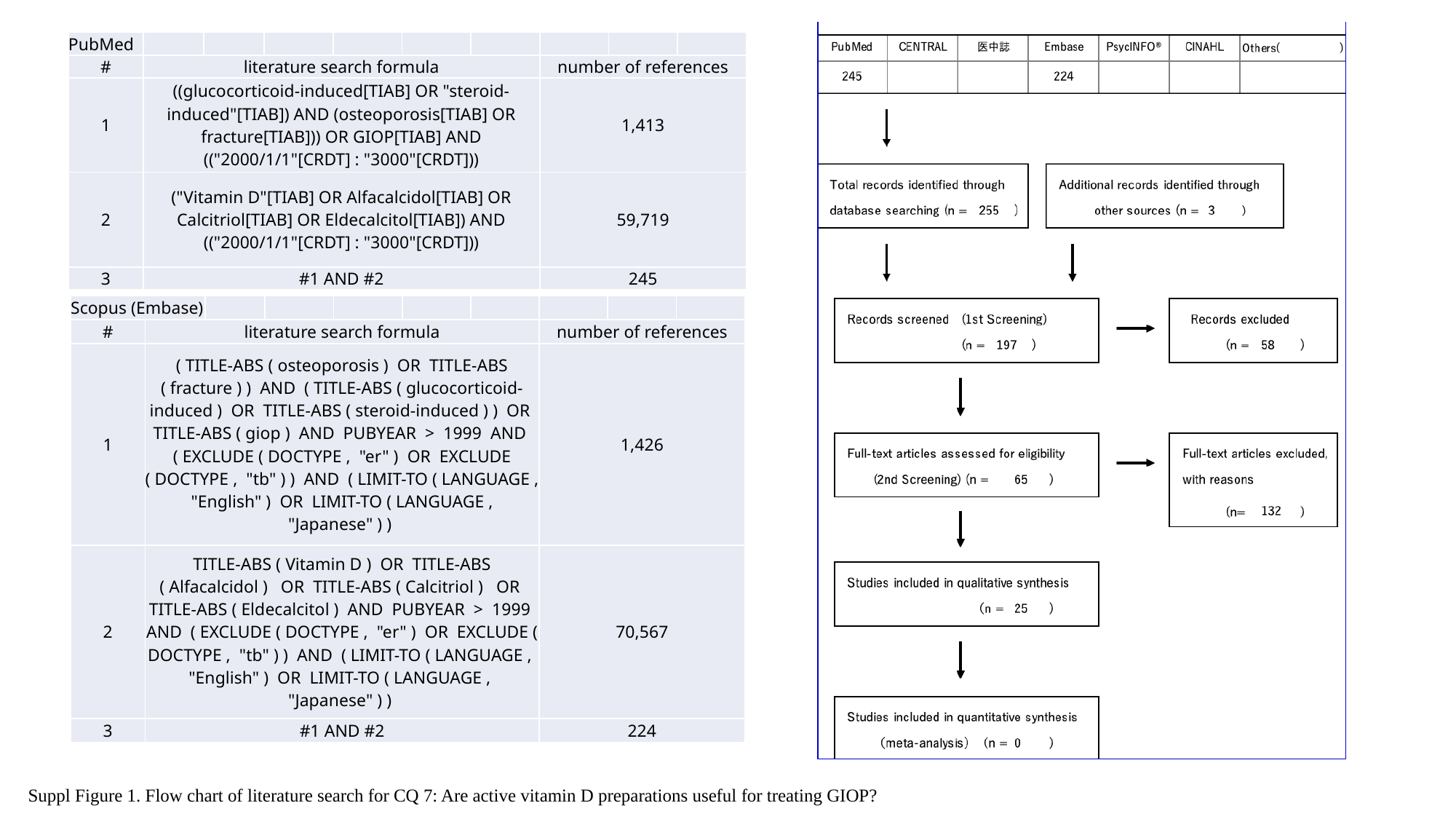

| PubMed | | | | | | | | | |
| --- | --- | --- | --- | --- | --- | --- | --- | --- | --- |
| # | literature search formula | | | | | | number of references | | |
| 1 | ((glucocorticoid-induced[TIAB] OR "steroid-induced"[TIAB]) AND (osteoporosis[TIAB] OR fracture[TIAB])) OR GIOP[TIAB] AND (("2000/1/1"[CRDT] : "3000"[CRDT])) | | | | | | 1,413 | | |
| 2 | ("Vitamin D"[TIAB] OR Alfacalcidol[TIAB] OR Calcitriol[TIAB] OR Eldecalcitol[TIAB]) AND (("2000/1/1"[CRDT] : "3000"[CRDT])) | | | | | | 59,719 | | |
| 3 | #1 AND #2 | | | | | | 245 | | |
| Scopus (Embase) | | | | | | | | | |
| --- | --- | --- | --- | --- | --- | --- | --- | --- | --- |
| # | literature search formula | | | | | | number of references | | |
| 1 | ( TITLE-ABS ( osteoporosis ) OR TITLE-ABS ( fracture ) ) AND ( TITLE-ABS ( glucocorticoid-induced ) OR TITLE-ABS ( steroid-induced ) ) OR TITLE-ABS ( giop ) AND PUBYEAR > 1999 AND ( EXCLUDE ( DOCTYPE , "er" ) OR EXCLUDE ( DOCTYPE , "tb" ) ) AND ( LIMIT-TO ( LANGUAGE , "English" ) OR LIMIT-TO ( LANGUAGE , "Japanese" ) ) | | | | | | 1,426 | | |
| 2 | TITLE-ABS ( Vitamin D ) OR TITLE-ABS ( Alfacalcidol ) OR TITLE-ABS ( Calcitriol ) OR TITLE-ABS ( Eldecalcitol ) AND PUBYEAR > 1999 AND ( EXCLUDE ( DOCTYPE , "er" ) OR EXCLUDE ( DOCTYPE , "tb" ) ) AND ( LIMIT-TO ( LANGUAGE , "English" ) OR LIMIT-TO ( LANGUAGE , "Japanese" ) ) | | | | | | 70,567 | | |
| 3 | #1 AND #2 | | | | | | 224 | | |
Suppl Figure 1. Flow chart of literature search for CQ 7: Are active vitamin D preparations useful for treating GIOP?

## Slide 2
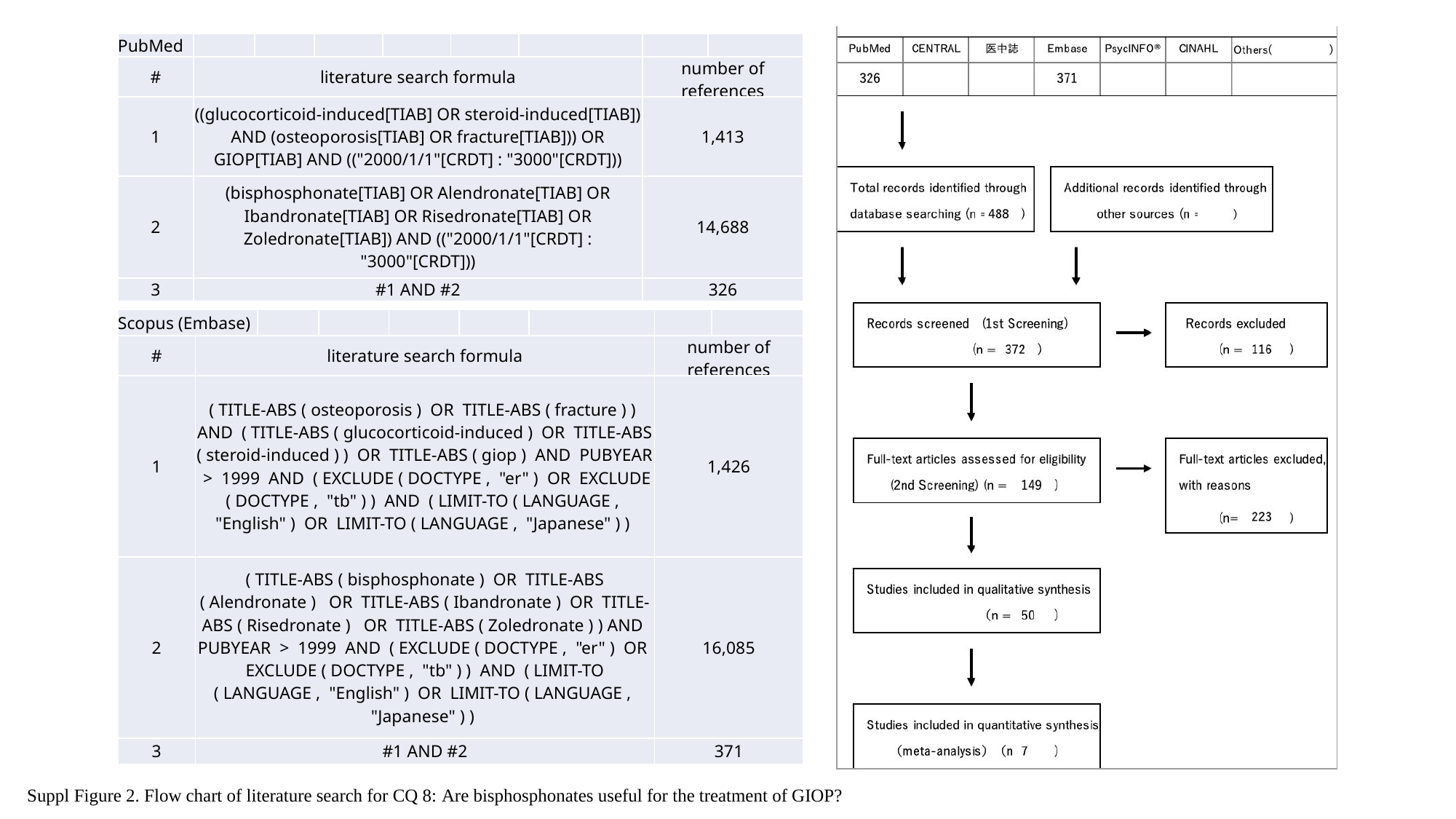

| PubMed | | | | | | | | |
| --- | --- | --- | --- | --- | --- | --- | --- | --- |
| # | literature search formula | | | | | | number of references | |
| 1 | ((glucocorticoid-induced[TIAB] OR steroid-induced[TIAB]) AND (osteoporosis[TIAB] OR fracture[TIAB])) OR GIOP[TIAB] AND (("2000/1/1"[CRDT] : "3000"[CRDT])) | | | | | | 1,413 | |
| 2 | (bisphosphonate[TIAB] OR Alendronate[TIAB] OR Ibandronate[TIAB] OR Risedronate[TIAB] OR Zoledronate[TIAB]) AND (("2000/1/1"[CRDT] : "3000"[CRDT])) | | | | | | 14,688 | |
| 3 | #1 AND #2 | | | | | | 326 | |
| Scopus (Embase) | | | | | | | | |
| --- | --- | --- | --- | --- | --- | --- | --- | --- |
| # | literature search formula | | | | | | number of references | |
| 1 | ( TITLE-ABS ( osteoporosis ) OR TITLE-ABS ( fracture ) ) AND ( TITLE-ABS ( glucocorticoid-induced ) OR TITLE-ABS ( steroid-induced ) ) OR TITLE-ABS ( giop ) AND PUBYEAR > 1999 AND ( EXCLUDE ( DOCTYPE , "er" ) OR EXCLUDE ( DOCTYPE , "tb" ) ) AND ( LIMIT-TO ( LANGUAGE , "English" ) OR LIMIT-TO ( LANGUAGE , "Japanese" ) ) | | | | | | 1,426 | |
| 2 | ( TITLE-ABS ( bisphosphonate ) OR TITLE-ABS ( Alendronate ) OR TITLE-ABS ( Ibandronate ) OR TITLE-ABS ( Risedronate ) OR TITLE-ABS ( Zoledronate ) ) AND PUBYEAR > 1999 AND ( EXCLUDE ( DOCTYPE , "er" ) OR EXCLUDE ( DOCTYPE , "tb" ) ) AND ( LIMIT-TO ( LANGUAGE , "English" ) OR LIMIT-TO ( LANGUAGE , "Japanese" ) ) | | | | | | 16,085 | |
| 3 | #1 AND #2 | | | | | | 371 | |
Suppl Figure 2. Flow chart of literature search for CQ 8: Are bisphosphonates useful for the treatment of GIOP?

## Slide 3
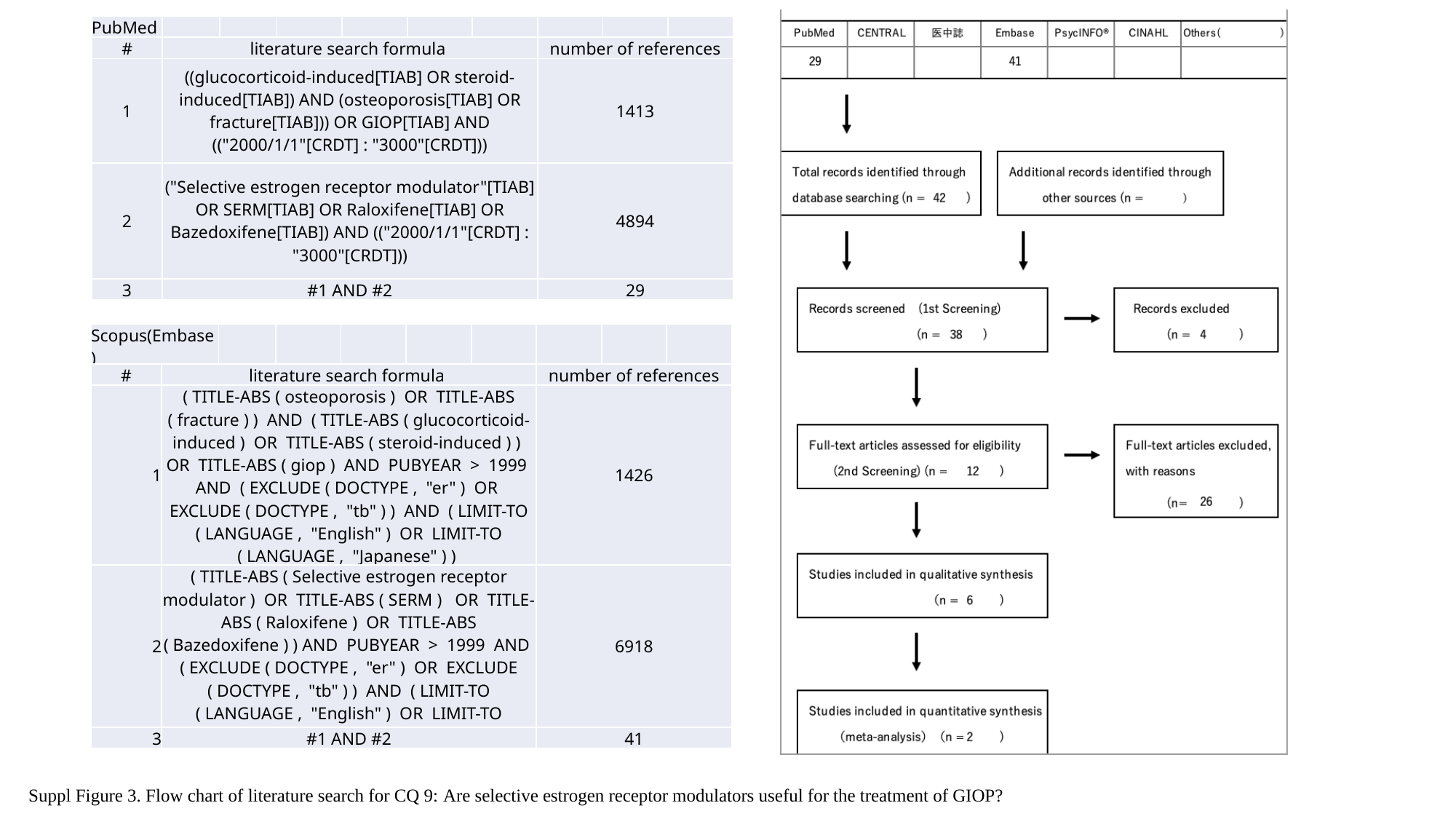

| PubMed | | | | | | | | | |
| --- | --- | --- | --- | --- | --- | --- | --- | --- | --- |
| # | literature search formula | | | | | | number of references | | |
| 1 | ((glucocorticoid-induced[TIAB] OR steroid-induced[TIAB]) AND (osteoporosis[TIAB] OR fracture[TIAB])) OR GIOP[TIAB] AND (("2000/1/1"[CRDT] : "3000"[CRDT])) | | | | | | 1413 | | |
| 2 | ("Selective estrogen receptor modulator"[TIAB] OR SERM[TIAB] OR Raloxifene[TIAB] OR Bazedoxifene[TIAB]) AND (("2000/1/1"[CRDT] : "3000"[CRDT])) | | | | | | 4894 | | |
| 3 | #1 AND #2 | | | | | | 29 | | |
| Scopus(Embase) | | | | | | | | | |
| --- | --- | --- | --- | --- | --- | --- | --- | --- | --- |
| # | literature search formula | | | | | | number of references | | |
| 1 | ( TITLE-ABS ( osteoporosis ) OR TITLE-ABS ( fracture ) ) AND ( TITLE-ABS ( glucocorticoid-induced ) OR TITLE-ABS ( steroid-induced ) ) OR TITLE-ABS ( giop ) AND PUBYEAR > 1999 AND ( EXCLUDE ( DOCTYPE , "er" ) OR EXCLUDE ( DOCTYPE , "tb" ) ) AND ( LIMIT-TO ( LANGUAGE , "English" ) OR LIMIT-TO ( LANGUAGE , "Japanese" ) ) | | | | | | 1426 | | |
| 2 | ( TITLE-ABS ( Selective estrogen receptor modulator ) OR TITLE-ABS ( SERM ) OR TITLE-ABS ( Raloxifene ) OR TITLE-ABS ( Bazedoxifene ) ) AND PUBYEAR > 1999 AND ( EXCLUDE ( DOCTYPE , "er" ) OR EXCLUDE ( DOCTYPE , "tb" ) ) AND ( LIMIT-TO ( LANGUAGE , "English" ) OR LIMIT-TO ( LANGUAGE , "Japanese" ) ) | | | | | | 6918 | | |
| 3 | #1 AND #2 | | | | | | 41 | | |
Suppl Figure 3. Flow chart of literature search for CQ 9: Are selective estrogen receptor modulators useful for the treatment of GIOP?

## Slide 4
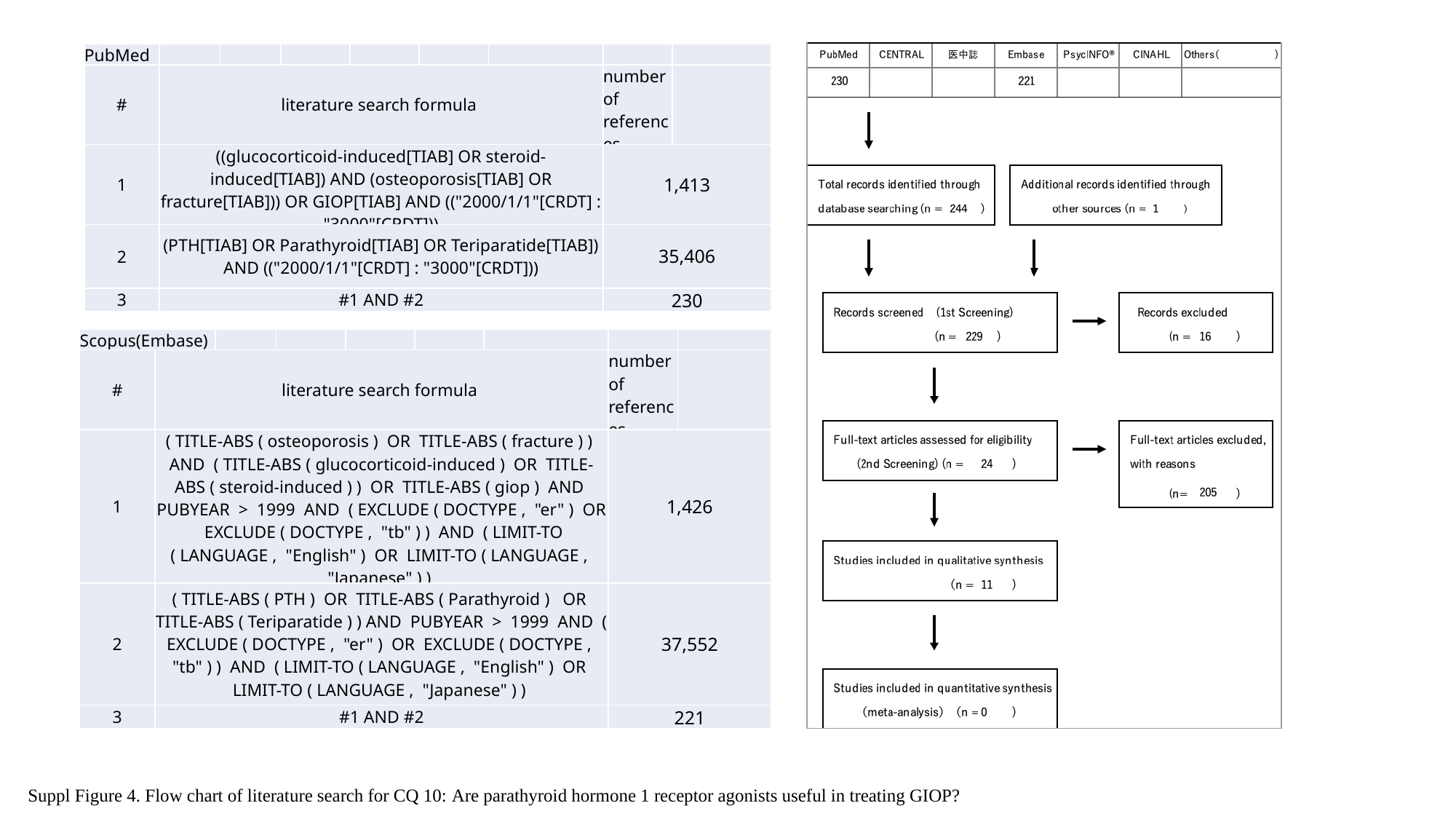

| PubMed | | | | | | | | |
| --- | --- | --- | --- | --- | --- | --- | --- | --- |
| # | literature search formula | | | | | | number of references | |
| 1 | ((glucocorticoid-induced[TIAB] OR steroid-induced[TIAB]) AND (osteoporosis[TIAB] OR fracture[TIAB])) OR GIOP[TIAB] AND (("2000/1/1"[CRDT] : "3000"[CRDT])) | | | | | | 1,413 | |
| 2 | (PTH[TIAB] OR Parathyroid[TIAB] OR Teriparatide[TIAB]) AND (("2000/1/1"[CRDT] : "3000"[CRDT])) | | | | | | 35,406 | |
| 3 | #1 AND #2 | | | | | | 230 | |
| Scopus(Embase) | | | | | | | | |
| --- | --- | --- | --- | --- | --- | --- | --- | --- |
| # | literature search formula | | | | | | number of references | |
| 1 | ( TITLE-ABS ( osteoporosis ) OR TITLE-ABS ( fracture ) ) AND ( TITLE-ABS ( glucocorticoid-induced ) OR TITLE-ABS ( steroid-induced ) ) OR TITLE-ABS ( giop ) AND PUBYEAR > 1999 AND ( EXCLUDE ( DOCTYPE , "er" ) OR EXCLUDE ( DOCTYPE , "tb" ) ) AND ( LIMIT-TO ( LANGUAGE , "English" ) OR LIMIT-TO ( LANGUAGE , "Japanese" ) ) | | | | | | 1,426 | |
| 2 | ( TITLE-ABS ( PTH ) OR TITLE-ABS ( Parathyroid ) OR TITLE-ABS ( Teriparatide ) ) AND PUBYEAR > 1999 AND ( EXCLUDE ( DOCTYPE , "er" ) OR EXCLUDE ( DOCTYPE , "tb" ) ) AND ( LIMIT-TO ( LANGUAGE , "English" ) OR LIMIT-TO ( LANGUAGE , "Japanese" ) ) | | | | | | 37,552 | |
| 3 | #1 AND #2 | | | | | | 221 | |
Suppl Figure 4. Flow chart of literature search for CQ 10: Are parathyroid hormone 1 receptor agonists useful in treating GIOP?

## Slide 5
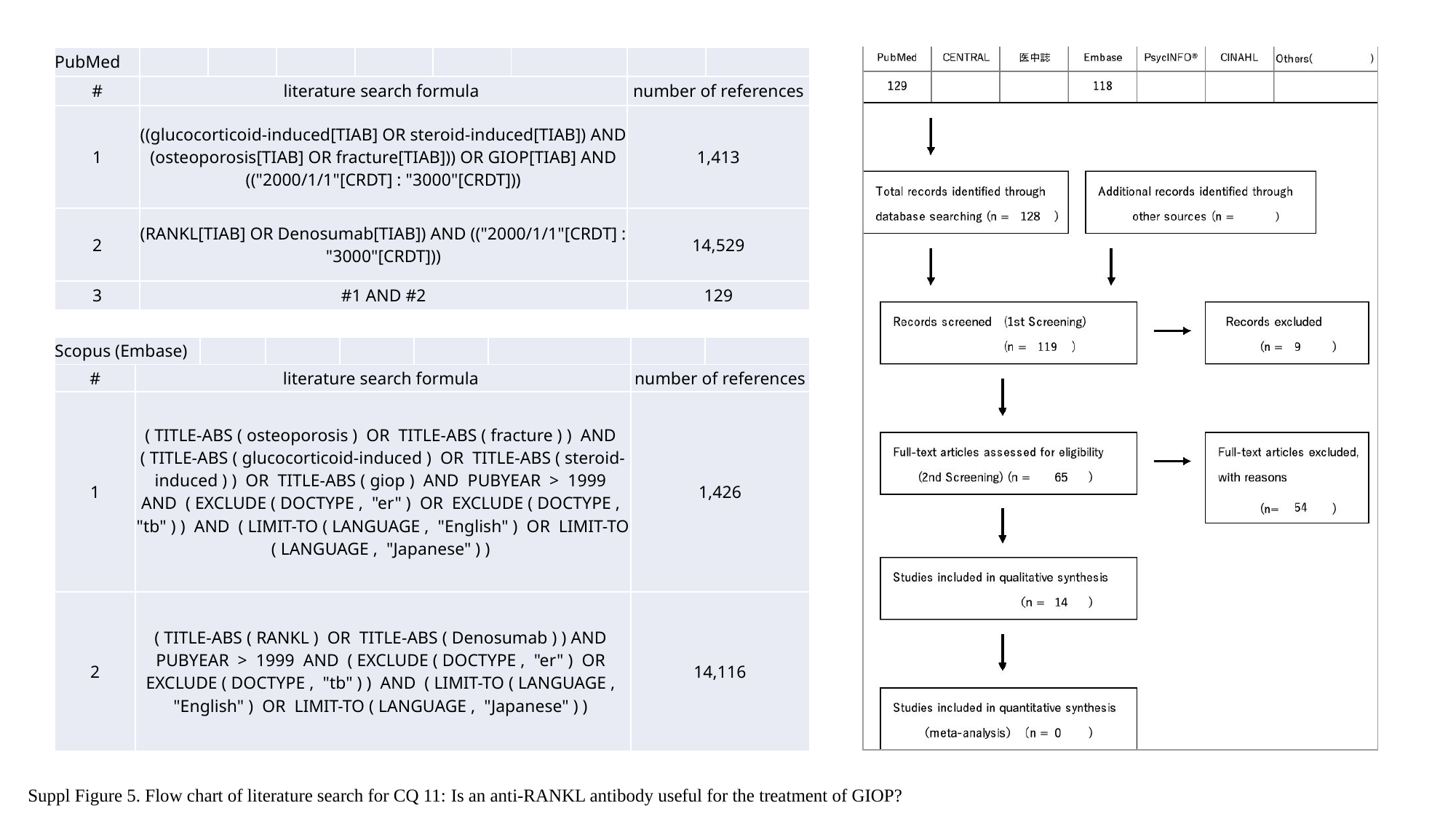

| PubMed | | | | | | | | |
| --- | --- | --- | --- | --- | --- | --- | --- | --- |
| # | literature search formula | | | | | | number of references | |
| 1 | ((glucocorticoid-induced[TIAB] OR steroid-induced[TIAB]) AND (osteoporosis[TIAB] OR fracture[TIAB])) OR GIOP[TIAB] AND (("2000/1/1"[CRDT] : "3000"[CRDT])) | | | | | | 1,413 | |
| 2 | (RANKL[TIAB] OR Denosumab[TIAB]) AND (("2000/1/1"[CRDT] : "3000"[CRDT])) | | | | | | 14,529 | |
| 3 | #1 AND #2 | | | | | | 129 | |
| Scopus (Embase) | | | | | | | | |
| --- | --- | --- | --- | --- | --- | --- | --- | --- |
| # | literature search formula | | | | | | number of references | |
| 1 | ( TITLE-ABS ( osteoporosis ) OR TITLE-ABS ( fracture ) ) AND ( TITLE-ABS ( glucocorticoid-induced ) OR TITLE-ABS ( steroid-induced ) ) OR TITLE-ABS ( giop ) AND PUBYEAR > 1999 AND ( EXCLUDE ( DOCTYPE , "er" ) OR EXCLUDE ( DOCTYPE , "tb" ) ) AND ( LIMIT-TO ( LANGUAGE , "English" ) OR LIMIT-TO ( LANGUAGE , "Japanese" ) ) | | | | | | 1,426 | |
| 2 | ( TITLE-ABS ( RANKL ) OR TITLE-ABS ( Denosumab ) ) AND PUBYEAR > 1999 AND ( EXCLUDE ( DOCTYPE , "er" ) OR EXCLUDE ( DOCTYPE , "tb" ) ) AND ( LIMIT-TO ( LANGUAGE , "English" ) OR LIMIT-TO ( LANGUAGE , "Japanese" ) ) | | | | | | 14,116 | |
Suppl Figure 5. Flow chart of literature search for CQ 11: Is an anti-RANKL antibody useful for the treatment of GIOP?

## Slide 6
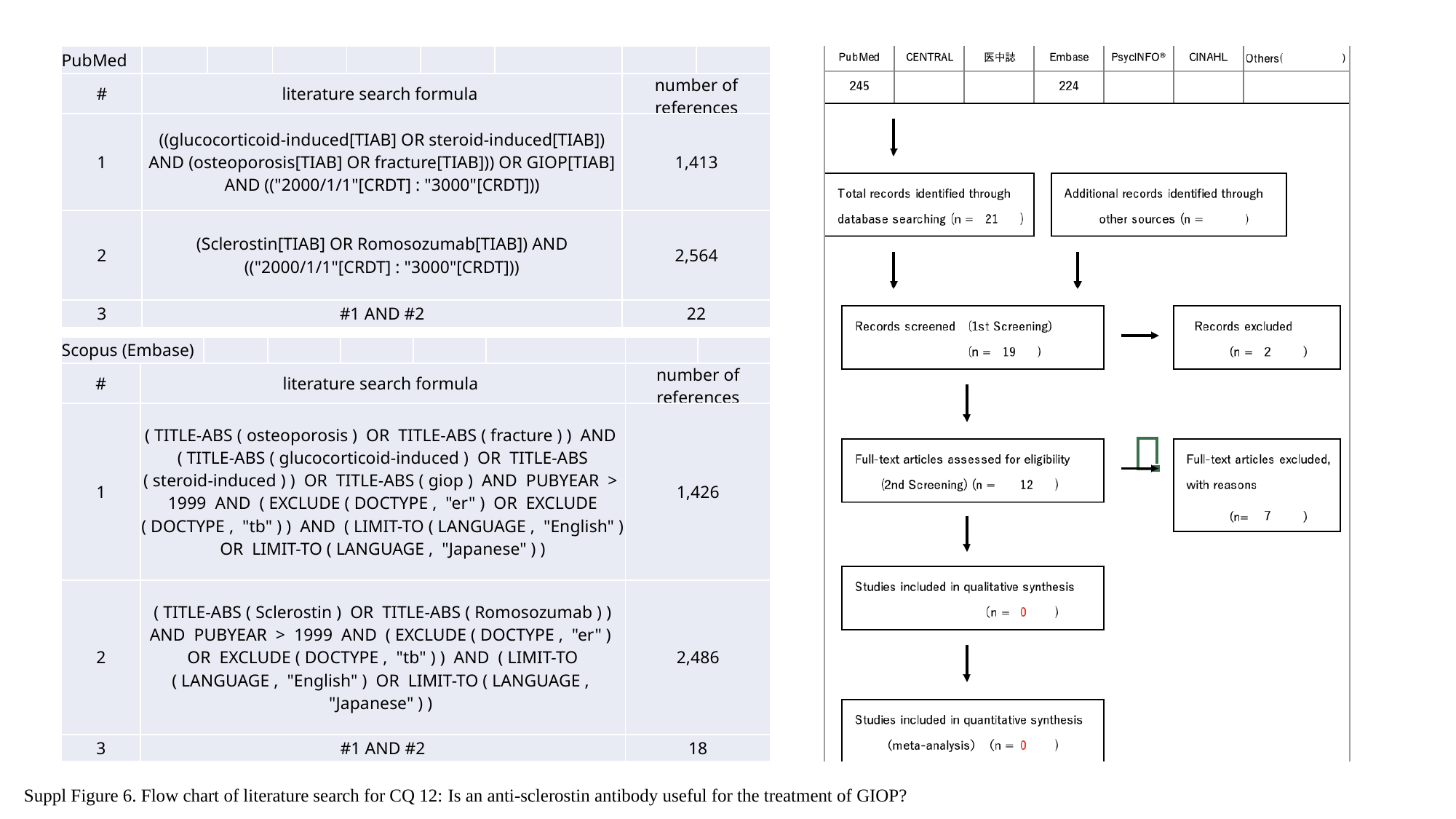

| PubMed | | | | | | | | |
| --- | --- | --- | --- | --- | --- | --- | --- | --- |
| # | literature search formula | | | | | | number of references | |
| 1 | ((glucocorticoid-induced[TIAB] OR steroid-induced[TIAB]) AND (osteoporosis[TIAB] OR fracture[TIAB])) OR GIOP[TIAB] AND (("2000/1/1"[CRDT] : "3000"[CRDT])) | | | | | | 1,413 | |
| 2 | (Sclerostin[TIAB] OR Romosozumab[TIAB]) AND (("2000/1/1"[CRDT] : "3000"[CRDT])) | | | | | | 2,564 | |
| 3 | #1 AND #2 | | | | | | 22 | |
| Scopus (Embase) | | | | | | | | |
| --- | --- | --- | --- | --- | --- | --- | --- | --- |
| # | literature search formula | | | | | | number of references | |
| 1 | ( TITLE-ABS ( osteoporosis ) OR TITLE-ABS ( fracture ) ) AND ( TITLE-ABS ( glucocorticoid-induced ) OR TITLE-ABS ( steroid-induced ) ) OR TITLE-ABS ( giop ) AND PUBYEAR > 1999 AND ( EXCLUDE ( DOCTYPE , "er" ) OR EXCLUDE ( DOCTYPE , "tb" ) ) AND ( LIMIT-TO ( LANGUAGE , "English" ) OR LIMIT-TO ( LANGUAGE , "Japanese" ) ) | | | | | | 1,426 | |
| 2 | ( TITLE-ABS ( Sclerostin ) OR TITLE-ABS ( Romosozumab ) ) AND PUBYEAR > 1999 AND ( EXCLUDE ( DOCTYPE , "er" ) OR EXCLUDE ( DOCTYPE , "tb" ) ) AND ( LIMIT-TO ( LANGUAGE , "English" ) OR LIMIT-TO ( LANGUAGE , "Japanese" ) ) | | | | | | 2,486 | |
| 3 | #1 AND #2 | | | | | | 18 | |
Suppl Figure 6. Flow chart of literature search for CQ 12: Is an anti-sclerostin antibody useful for the treatment of GIOP?

## Slide 7
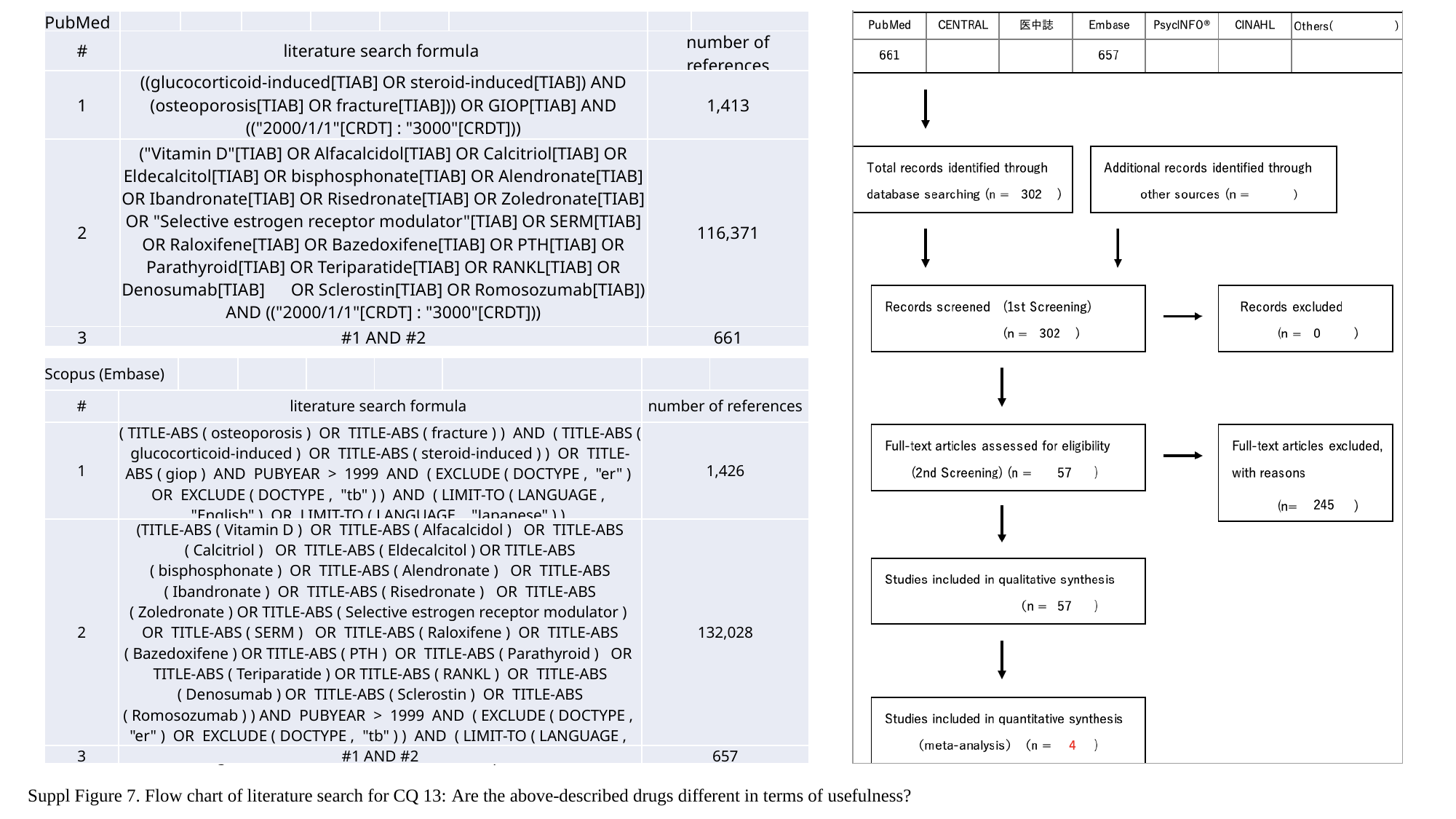

| PubMed | | | | | | | | |
| --- | --- | --- | --- | --- | --- | --- | --- | --- |
| # | literature search formula | | | | | | number of references | |
| 1 | ((glucocorticoid-induced[TIAB] OR steroid-induced[TIAB]) AND (osteoporosis[TIAB] OR fracture[TIAB])) OR GIOP[TIAB] AND (("2000/1/1"[CRDT] : "3000"[CRDT])) | | | | | | 1,413 | |
| 2 | ("Vitamin D"[TIAB] OR Alfacalcidol[TIAB] OR Calcitriol[TIAB] OR Eldecalcitol[TIAB] OR bisphosphonate[TIAB] OR Alendronate[TIAB] OR Ibandronate[TIAB] OR Risedronate[TIAB] OR Zoledronate[TIAB] OR "Selective estrogen receptor modulator"[TIAB] OR SERM[TIAB] OR Raloxifene[TIAB] OR Bazedoxifene[TIAB] OR PTH[TIAB] OR Parathyroid[TIAB] OR Teriparatide[TIAB] OR RANKL[TIAB] OR Denosumab[TIAB]　OR Sclerostin[TIAB] OR Romosozumab[TIAB]) AND (("2000/1/1"[CRDT] : "3000"[CRDT])) | | | | | | 116,371 | |
| 3 | #1 AND #2 | | | | | | 661 | |
| Scopus (Embase) | | | | | | | | |
| --- | --- | --- | --- | --- | --- | --- | --- | --- |
| # | literature search formula | | | | | | number of references | |
| 1 | ( TITLE-ABS ( osteoporosis ) OR TITLE-ABS ( fracture ) ) AND ( TITLE-ABS ( glucocorticoid-induced ) OR TITLE-ABS ( steroid-induced ) ) OR TITLE-ABS ( giop ) AND PUBYEAR > 1999 AND ( EXCLUDE ( DOCTYPE , "er" ) OR EXCLUDE ( DOCTYPE , "tb" ) ) AND ( LIMIT-TO ( LANGUAGE , "English" ) OR LIMIT-TO ( LANGUAGE , "Japanese" ) ) | | | | | | 1,426 | |
| 2 | (TITLE-ABS ( Vitamin D ) OR TITLE-ABS ( Alfacalcidol ) OR TITLE-ABS ( Calcitriol ) OR TITLE-ABS ( Eldecalcitol ) OR TITLE-ABS ( bisphosphonate ) OR TITLE-ABS ( Alendronate ) OR TITLE-ABS ( Ibandronate ) OR TITLE-ABS ( Risedronate ) OR TITLE-ABS ( Zoledronate ) OR TITLE-ABS ( Selective estrogen receptor modulator ) OR TITLE-ABS ( SERM ) OR TITLE-ABS ( Raloxifene ) OR TITLE-ABS ( Bazedoxifene ) OR TITLE-ABS ( PTH ) OR TITLE-ABS ( Parathyroid ) OR TITLE-ABS ( Teriparatide ) OR TITLE-ABS ( RANKL ) OR TITLE-ABS ( Denosumab ) OR TITLE-ABS ( Sclerostin ) OR TITLE-ABS ( Romosozumab ) ) AND PUBYEAR > 1999 AND ( EXCLUDE ( DOCTYPE , "er" ) OR EXCLUDE ( DOCTYPE , "tb" ) ) AND ( LIMIT-TO ( LANGUAGE , "English" ) OR LIMIT-TO ( LANGUAGE , "Japanese" ) ) | | | | | | 132,028 | |
| 3 | #1 AND #2 | | | | | | 657 | |
Suppl Figure 7. Flow chart of literature search for CQ 13: Are the above-described drugs different in terms of usefulness?
