# Supplementary material for: The 2023 Guidelines for the management and treatment of glucocorticoid-induced osteoporosis
Source: J Bone Miner Metab. 2024 Mar 28;42(2):143–54. doi: 10.1007/s00774-024-01502-w (PMC10982086; doi:10.1007/s00774-024-01502-w)
Supplement: Supplementary file 2 — Supplementary file2 (DOCX 41 KB) [file 774_2024_1502_MOESM2_ESM.docx]

Supplemental data 1. List of references used in the systematic literature review for each clinical question.

1. Saag KG. Glucocorticoid-induced osteoporosis. Endocrinol Metab Clin N Am 32 (2003) 135–157
2. McDonough AK, Curtis JR, Saag KG. The epidemiology of glucocorticoid-associated adverse events. Curr Opin Rheumatol. 2008 Mar;20(2):131-7
3. Raterman HG, Bultink IEM, Lems WF. Current Treatments and New Developments in the Management of Glucocorticoid-induced Osteoporosis. Drugs. 2019 Jul;79(10):1065-1087
4. Chiodini I, Falchetti A, Merlotti D, et al. Updates in epidemiology, pathophysiology and management strategies of glucocorticoid-induced osteoporosis. Expert Rev Endocrinol Metab 2020; 15: 283-298
5. Chotiyarnwong P, McCloskey EV. Pathogenesis of glucocorticoid-induced osteoporosis and options for treatment. Nat Rev Endocrinol (2020) 16: 437-447
6. Ebeling PR, Nguyen HH, Aleksova J, Vincent AJ, Wong P, Milat F. Secondary Osteoporosis. Endocr Rev. 2022 Mar 9;43(2):240-313
7. Buckley L, Guyatt G, Fink HA, et al. 2017 American College of Rheumatology Guideline for the Prevention and Treatment of Glucocorticoid-Induced Osteoporosis. Arthritis Rheumatol. 2017; 69: 1521-1537
8. Nawata H, Soen S, Takayanagi R, et al; The Subcommittee to Study Diagnostic Criteria for Glucocorticoid-Induced Osteoporosis. Guidelines on the management and treatment of glucocorticoid-induced osteoporosis of the Japanese Society for Bone and Mineral Research 2004. J Bone Miner Metab 23: 105-9, 2005
9. Suzuki Y, Nawata H, Soen S, et al. Guidelines on the management and treatment of glucocorticoid-induced osteoporosis of the Japanese society for bone and mineral research: 2014 update. J Bone Miner Metab (2014) 32: 337-50
10. Díez-Pérez A, Hooven FH, Adachi JD, Adami S, Anderson FA, et al. Regional differences in treatment for osteoporosis. The Global Longitudinal Study of Osteoporosis in Women (GLOW). Bone. 2011 Sep;49(3):493-8
11. Soen S, Kaku M, Okubo N, Touzeni S, Saito K, Kobayashi M. Epidemiology of glucocorticoid-induced osteoporosis and management of associated fracture risk in Japan. J Bone Miner Metab. 2021 Nov;39(6):1019-1030
12. Iki M, Fujimori K, Nakatoh S, Tamaki J, Ishii S, Okimoto N, Kamiya K, Ogawa S. Guideline adherence by physicians for management of glucocorticoid-induced osteoporosis in Japan: a nationwide health insurance claims database study. Osteoporos Int. 2022 May;33(5):1097-1108
13. Overman RA, Yeh JY, Deal CL. Prevalence of oral glucocorticoid usage in the United States: a general population perspective. Arthritis Care Res (Hoboken) 2013;65:294–8
14. Fardet L, Petersen I, Nazareth I. Prevalence of long-term oral glucocorticoid prescriptions in the UK over the past 20 years. Rheumatology (Oxford) 2011;50:1982–90
15. Gudbjornsson B, Juliusson UI, Gudjonsson FV. Prevalence of long term steroid treatment and the frequency of decision making to prevent steroid induced osteoporosis in daily clinical practice. Ann Rheum Dis 2002;61:32–6
16. Trijau S, de Lamotte G, Pradel V, Natali F, Allaria-Lapierre V, Coudert H, Pham T, Sciortino V, Lafforgue P. Osteoporosis prevention among chronic glucocorticoid users: results from a public health insurance database. RMD Open. 2016 Jul 7;2(2):e000249
17. van Staa TP, Leufkens HGM, Cooper C. The Epidemiology of Corticosteroid-Induced Osteoporosis: A Meta-Analysis. Osteoporos Int (2002) 13:777–87
18. Steinbuch M, Youket TE, Cohen S. Oral glucocorticoid use is associated with an increased risk of fracture. Osteoporos Int. 2004 Apr;15(4):323-8
19. De Vries F, Bracke M, Leufkens HG, Lammers JW, Cooper C, Van Staa TP. Fracture risk with intermittent high-dose oral glucocorticoid therapy. Arthritis Rheum. 2007 Jan;56(1):208-14
20. Abtahi S, Driessen JHM, Burden AM, Souverein PC, van den Bergh JP, van Staa TP, Boonen A, de Vries F. Low-dose oral glucocorticoid therapy and risk of osteoporotic fractures in patients with rheumatoid arthritis: a cohort study using the Clinical Practice Research Datalink. Rheumatology (Oxford). 2022 Apr 11;61(4):1448-1458
21. Amiche MA, Abtahi S, Driessen JHM, Vestergaard P, de Vries F, Cadarette SM, Burden AM. Impact of cumulative exposure to high-dose oral glucocorticoids on fracture risk in Denmark: a population-based case-control study. Arch Osteoporos. 2018 Mar 18;13(1):30
22. Suzuki Y, Nawata H, Soen S, Fujiwara S, Nakayama H, Tanaka I, Ozono K, Sagawa A, Takayanagi R, Tanaka H, Miki T, Masunari N, Tanaka Y (2014) Guidelines on the management and treatment of glucocorticoid-induced osteoporosis of the Japanese Society for Bone and Mineral Research: 2014 update. J Bone Miner Metab 32:337-50 doi:10.1007/s00774-014-0586-6
23. Soen S, Kaku M, Okubo N, Touzeni S, Saito K, Kobayashi M (2021) Epidemiology of glucocorticoid-induced osteoporosis and management of associated fracture risk in Japan. J Bone Miner Metab 39:1019-30 doi:10.1007/s00774-021-01236-z
24. Shinoda K, Taki H (2021) Treatment of Glucocorticoid-Induced Osteoporosis and Risk Factors for New Vertebral Fractures in Female Patients with Autoimmune Diseases. J Osteoporos 2021:5515653 doi:10.1155/2021/5515653
25. Florez H, Hernandez-Rodriguez J, Muxi A, Carrasco JL, Prieto-Gonzalez S, Cid MC, Espinosa G, Gomez-Puerta JA, Monegal A, Guanabens N, Peris P (2020) Trabecular bone score improves fracture risk assessment in glucocorticoid-induced osteoporosis. Rheumatology (Oxford) 59:1574-80 doi:10.1093/rheumatology/kez464
26. Florez H, Hernandez-Rodriguez J, Carrasco JL, Prieto-Gonzalez S, Muxi A, Filella X, Ruiz-Gaspa S, Gomez-Puerta JA, Cid M, Espinosa G, Monegal A, Guanabens N, Peris P (2020) Vertebral fracture risk in glucocorticoid-induced osteoporosis: the role of hypogonadism and corticosteroid boluses. RMD Open 6 doi:10.1136/rmdopen-2020-001355
27. Mori Y, Izumiyama T, Baba K, Mori N, Fujii H, Ishii T, Itoi E (2020) Evaluation of risk factors of vertebral fracture in Japanese female patients with glucocorticoid-induced osteoporosis. J Orthop Surg Res 15:290 doi:10.1186/s13018-020-01813-4
28. Che H, Breuil V, Cortet B, Paccou J, Thomas T, Chapuis L, Debiais F, Mehsen-Cetre N, Javier RM, Loiseau Peres S, Roux C, Briot K (2019) Vertebral fractures cascade: potential causes and risk factors. Osteoporos Int 30:555-63 doi:10.1007/s00198-018-4793-1
29. miche MA, Levesque LE, Gomes T, Adachi JD, Cadarette SM (2018) Effectiveness of Oral Bisphosphonates in Reducing Fracture Risk Among Oral Glucocorticoid Users: Three Matched Cohort Analyses. J Bone Miner Res 33:419-29 doi:10.1002/jbmr.3318
30. Buckley L, Guyatt G, Fink HA, Cannon M, Grossman J et al. (2017) 2017 American College of Rheumatology Guideline for the Prevention and Treatment of Glucocorticoid-Induced Osteoporosis. Arthritis Rheumatol 69:1521-37 doi:10.1002/art.40137
31. Ma CC, Xu SQ, Gong X, Wu Y, Qi S, Liu W, Xu JH (2017) Prevalence and risk factors associated with glucocorticoid-induced osteoporosis in Chinese patients with rheumatoid arthritis. Arch Osteoporos 12:33 doi:10.1007/s11657-017-0329-0
32. Morin C, Fardet L (2015) Systemic glucocorticoid therapy: risk factors for reported adverse events and beliefs about the drug. A cross-sectional online survey of 820 patients. Clin Rheumatol 34:2119-26 doi:10.1007/s10067-015-2953-7
33. Saag KG.: Bone safety of low-dose glucocorticoids in rheumatic diseases. Ann. N. Y. Acad. Sci. 1318: 55-64, 2014
34. 2. Buckley L, Guyatt G, Fink HA, et al.: 2017 American College of Rheumatology Guideline for the prevention and treatment of glucocorticoid-induced osteoporosis. Arthritis Rheumatol. 69(8): 1521-1537, 2017
35. 3. Caplan A, Fett N, Rosenbach M, et al: Prevention and management of glucocorticoid-induced side effects: A comprehensive review: A review of glucocorticoid pharmacology and bone health. Journal of the American Academy of Dermatology 76:1-9, 2017
36. 4. Kageyama G, Okano T, Yamamoto Y, et: Very high frequency of fragility fractures associated with high-dose glucocorticoids in postmenopausal women: A retrospective study. Bone Rep 6:3-8, 2017
37. 5. Adami G, Saag KG: Glucocorticoid-induced osteoporosis: 2019 concise clinical review. Osteoporosis Int 30: 1145-1156, 2019
38. 6. Hu K, Adachi JD: Glucocorticoid induced osteoporosis. Expert Review of Endocrinology & Metabolism 14(4), 259-266, 2019
39. Rossini　M, Viapiana O, Vitiello M et al. Prevalence and incidence of osteoporotic fractures in patients on long-term glucocorticoid treatment for rheumatic diseases: the Glucocorticoid Induced OsTeoporosis TOol (GIOTTO) study. Reumatismo 2017;69:30-39.
40. Fujiwara S, Ishii S, Hamasaki T et al. Incidence of fractures among patients receiving medications for type 2 diabetes or chronic obstructive pulmonary disease and glucocorticoid users according to the National Claims Database in Japan　 Archives of Osteoporosis 2021;16:106　https://doi.org/10.1007/s11657-021-00968-9
41. Ward LM,　Ma J,　Lang B et al. Bone morbidity and recovery in children with acute lymphoblastic leukemia: Results of a six-year prospective cohort study. J Bone Miner Res 2018; 33:1435–1443　DOI: 10.1002/jbmr.3447
42. Huber AM, Gaboury D. A. Cabral DA et al. Prevalent vertebral fractures among children　initiating glucocorticoid therapy for the treatment of rheumatic disorders. Arthritis Care & Research　2010;62:516–526　DOI 10.1002/acr.20171
43. LeBlanc CMA, Ma J, Taljaard M et al. Incident vertebral fractures and risk factors in the first　three years following glucocorticoid initiation among pediatric patients with rheumatic disorders. J Bone Miner Res 2015; 30:1667–1675　DOI: 10.1002/jbmr.2511
44. Ward LM. Glucocorticoid-induced osteoporosis: Why kids are different? Frontiers in Endocrinology. 2020 11 article 578 <https://doi.org/10.3389/fendo.2020.00576>
45. Shevroja E、Cafarelli FP, Guglielmi G et al. DXA parameters, trabecular bone score (TBS) and bone mineral density (BMD), in fracture risk prediction in endocrine-mediated secondary osteoporosis. Endocrine 2021;74:20–28 https://doi.org/10.1007/s12020-021-02806-x
46. Lee YH, Woo JH, Choi SJ et al. Effects of low-dose corticosteroids on the bone mineral density of patients with rheumatoid arthritis: a meta-analysis,　J Investig Med 2008;56:1011-1018.
47. Siu B. Haraoui, R. Bissonnette, et al., Meta-analysis of tumor necrosis factor inhibitors and glucocorticoids on bone density in rheumatoid arthritis and ankylosing spondylitis trials, Arthritis Care Res. (Hoboken) 2015;67:754-764 <http://dx.doi.org/10.1002/acr.22519>.
48. Lems WF, Baak MME, van Tuy LHD et al. One-year effects of glucocorticoids on bone density: a meta-analysis in cohorts on high and low-dose therapy RMD Open 2016;2:e000313. doi:10.1136/rmdopen-2016-000313
49. Blavnsfeldta ABG, de Thuraha A, Thomsenb MD et al. The effect of glucocorticoids on bone mineral density in patients with rheumatoid arthritis: A systematic review and meta-analysis of randomized,　controlled trials　Bone 2018; 114:172-180.
50. Weber DR, Boyce A, Gordon C et al., The utility of DXA assessment at the forearm, proximal femur, and lateral distal femur, and vertebral fracture assessment in the pediatric population: 2019 ISCD Official Position　J Clin Densitom. 2019 ; 22: 567–589.　doi:10.1016/j.jocd.2019.07.002.
51. Buckley L, Guyatt G, Ha Fet al. 2017 American College of Rheumatology guideline for the prevention and treatment of glucocorticoid-induced osteoporosis Arthritis Care & Research 2017;69:1095–1110 DOI 10.1002/acr.23279
52. Herath M, Langdahl B, Ebeling PR et al. Challenges in the diagnosis and management of glucocorticoid‐induced osteoporosis in younger and older adults. Clinical Endocrinology. 2022;96:460–474.
53. Lee1 JH、Lee　YK, Oh　SH, et al.　A systematic review of diagnostic accuracy of vertebral fracture　assessment (VFA) in postmenopausal women and elderly men Osteoporos Int 2016; 27:1691–1699
54. Compston J. Management of glucocorticoid‐induced osteoporosis: What is new? 　Int J Rheum Dis. 2019;22:1595–1597.
55. Paggiosi MA, Peel FA, Eastell R　The impact of glucocorticoid therapy on trabecular bone score　in older women　Osteoporos Int 2015;26:1773–1780 DOI 10.1007/s00198-015-3078-1
56. Florez H, Herna´ ndez-Rodrı´guez　J, Muxi　A et al. Trabecular bone score improves fracture risk assessment in glucocorticoid-induced osteoporosis　Rheumatology 2020;59:15741580 doi:10.1093/rheumatology/kez464
57. Nowakowska-Płaza A, Wroński J, Sudoł-Szopińska I et al., Clinical utility of trabecular bone score (TBS) in fracture risk assessment of patients with rheumatic diseases treated with　glucocorticoids　Horm Metab Res 2021; 53: 499–503
58. Harvey　NC,　Glüer　CC, N. Binkley　N et al. Trabecular bone score (TBS) as a new complementary approach for osteoporosis evaluation in clinical practice:　A consensus report of a European Society for Clinical and Economic Aspects of Osteoporosis and Osteoarthritis (ESCEO) Working Group　　*Bone*. 2015; 78: 216–224. doi:10.1016/j.bone.2015.05.016.
59. Shevroja1 E, Cafarelli FP,　Guglielmi G et al.　DXA parameters, trabecular bone score (TBS) and bone mineral density (BMD), in fracture risk prediction in endocrine-mediated　secondary osteoporosis　Endocrine 2021;74:20–28　<https://doi.org/10.1007/s12020-021-02806-x>
60. Adami1 G, Saag KG. Glucocorticoid-induced osteoporosis: 2019 concise clinical review. Osteoporosis International 2019;30:1145–1156 <https://doi.org/10.1007/s00198-019-04906-x>
61. Saag KG,　Agnusdei　D, Hans D　et al. Trabecular bone score in patients with chronic glucocorticoid therapy–induced osteoporosis treated with Alendronate or Teriparatide　Arthritis Rheumatology 2016;68:2122–2128　DOI 10.1002/art.39726
62. Sutter S, Nishiyama KK, Kepley A et al. Abnormalities in cortical bone, trabecular plates, and stiffness in postmenopausal women treated with glucocorticoids.　Clin Endo Metab 2014; 99:4231–4240.
63. Tang XL, Qin L, Kwok AW et al. Alterations of bone geometry, density, microarchitecture,　and biomechanical properties in systemic lupus erythematosus　on long-term glucocorticoid: a case–control study　using HR-pQCT　Osteoporos Int 2013;24:1817–1826　DOI 10.1007/s00198-012-2177-5
64. Graeff C, Marin F, Petto H et al. High resolution quantitative computed tomography-based assessment of trabecular　microstructure and strength estimates by finite-element analysis of the spine, but not DXA, reflects vertebral fracture status in men with　glucocorticoid-induced osteoporosis　Bone 2013;52:568-577
65. Compston J Glucocorticoid-induced osteoporosis :an update. Endocrine 2018;61:4-16.
66. [Recommendations for the prevention and treatment of glucocorticoid-induced osteoporosis: 2001 update. American College of Rheumatology Ad Hoc Committee on Glucocorticoid-Induced Osteoporosis.](https://pubmed.ncbi.nlm.nih.gov/11465699/) Arthritis Rheum. 2001 Jul;44(7):1496-503.
67. [2017 American College of Rheumatology Guideline for the Prevention and Treatment of Glucocorticoid-Induced Osteoporosis.](https://pubmed.ncbi.nlm.nih.gov/28585410/) Buckley L, Guyatt G, Fink HA, Cannon M, Grossman J, Hansen KE, Humphrey MB, Lane NE, Magrey M, Miller M, Morrison L, Rao M, Byun Robinson A, Saha S, Wolver S, Bannuru RR, Vaysbrot E, Osani M, Turgunbaev M, Miller AS, McAlindon T.Arthritis Care Res (Hoboken). 2017 Aug;69(8):1095-1110.
68. [Sylvie Huybers](https://pubmed.ncbi.nlm.nih.gov/?term=Huybers+S&cauthor_id=16901990) [^1^](https://pubmed.ncbi.nlm.nih.gov/16901990/#affiliation-1), [Ton H J Naber](https://pubmed.ncbi.nlm.nih.gov/?term=Naber+TH&cauthor_id=16901990), [René J M Bindels](https://pubmed.ncbi.nlm.nih.gov/?term=Bindels+RJ&cauthor_id=16901990), [Joost G J Hoenderop](https://pubmed.ncbi.nlm.nih.gov/?term=Hoenderop+JG&cauthor_id=16901990) [Prednisolone-induced Ca2+ malabsorption is caused by diminished expression of the epithelial Ca2+ channel TRPV6.](https://pubmed.ncbi.nlm.nih.gov/16901990/) Am J Physiol Gastrointest Liver Physiol. 2007 Jan;292(1):G92-7. doi: 10.1152/ajpgi.00317.2006. Epub 2006 Aug 10.
69. Chotiyarnwong P, McCloskey EV [Pathogenesis of glucocorticoid-induced osteoporosis and options for treatment.](https://pubmed.ncbi.nlm.nih.gov/32286516/) Nat Rev Endocrinol. 2020 Aug;16(8):437-447.
70. Ross AC, Manson JE, Abrams SA, Aloia JF, Brannon PM, Clinton SK, et al. The 2011 report on dietary reference intakes for calcium and vitamin D from the Institute of Medicine: what clinicians need to know. J Clin Endocrinol Metab 2011;96:53–8.
71. Nawata H, Soen S, Takayanagi R, Tanaka I, Takaoka K, Fukunaga M, Matsumoto T, Suzuki Y, Tanaka H, Fujiwara S, Miki T, Sagawa A, Nishizawa Y, Seino Y [Guidelines on the management and treatment of glucocorticoid-induced osteoporosis of the Japanese Society for Bone and Mineral Research (2004).](https://pubmed.ncbi.nlm.nih.gov/15750687/); Subcommittee to Study Diagnostic Criteria for Glucocorticoid-Induced Osteoporosis.J Bone Miner Metab. 2005;23(2):105-9.
72. Suzuki Y, Nawata H, Soen S, Fujiwara S, Nakayama H, Tanaka I, Ozono K, Sagawa A, Takayanagi R, Tanaka H, Miki T, Masunari N, Tanaka Y.

[Guidelines on the management and treatment of glucocorticoid-inducedosteoporosis of the Japanese Society for Bone and Mineral Research: 2014 update.](https://pubmed.ncbi.nlm.nih.gov/24818875/) J Bone Miner Metab. 2014 Jul;32(4):337-50

1. Compston J. [Glucocorticoid-induced osteoporosis: an update.](https://pubmed.ncbi.nlm.nih.gov/29691807/) Endocrine 2018; 61:7-16
2. Compston J., Cooper A., Cooper C., Gittoes N., Gregson C., Harvey N., Hope S., Kanis J.A., McCloskey E.V., Poole K.E.S., Reid D.M., Selby P., Thompson F., Thurston A., Vine N., The National Osteoporosis Guideline Group (NOGG)　 UK clinical guideline for the prevention and treatment of osteoporosis　Archives of Osteoporosis 2017; 12:43-
3. EFSA Panel on Dietetic Products, Nutrition and Allergies (NDA). Scientific opinion on dietary reference values for calcium. EFSA J 2015;13. doi: 10.2903/j.efsa.2015.4101.
4. Institute of Medicine. Dietary reference intakes for calcium and vitamin D. Washington, DC: National Academies Press, 2011. <http://www.nap.edu/catalog/13050> (24 March 2022, date last accessed).
5. Dawson-Hughes B, Mithal A, Bonjour J-P. et al. IOF position statement: vitamin D recommendations for older adults. Osteoporos Int 2010;21:1151–4.
6. EFSA Panel on Dietetic Products, Nutrition and Allergies (NDA). Dietary reference values for vitamin D. EFSA J2016;14:e04547.
7. Kamen DL, Cooper GS, Bouali H. et al. Vitamin D deficiency in systemic lupus erythematosus. Autoimmun Rev2006;5:114–7.
8. LeBoff MS, Chou SH, Murata EM. et al. Effects of supplemental vitamin D on bone health outcomes in women and men in the VITamin D and OmegA‐3 TriaL (VITAL). J Bone Miner Res 2020;35:883–93.
9. Sambrook P, Birmingham J, Kelly P. et al. Prevention of corticosteroid osteoporosis – a comparison of calcium, calcitriol, and calcitonin. N Engl J Med 1993;328:1747–52.
10. Buckley LM, Leib ES, Cartularo KS, Vacek PM, Cooper SM.. Calcium and vitamin D3 supplementation prevents bone loss in the spine secondary to low-dose corticosteroids in patients with rheumatoid arthritis. Ann Intern Med 1996;125:961–8.
11. Amin S, LaValley MP, Simms RW, Felson DT.. The role of vitamin D in corticosteroid-induced osteoporosis: a meta-analytic approach. Arthritis Rheum 1999;42:1740–51.
12. Richy F, Ethgen O, Bruyere O, Reginster J-Y.. Efficacy of alphacalcidol and calcitriol in primary and corticosteroid-induced osteoporosis: a meta-analysis of their effects on bone mineral density and fracture rate. Osteoporos Int 2004;15:301–10.
13. Homik J, Suarez-Almazor ME, Shea B. et al. Calcium and vitamin D for corticosteroid-induced osteoporosis. Cochrane Database Syst Rev 1998. doi: 10.1002/14651858.CD000952.
14. Chen L., Shi X., Weng S.-J., Xie J., Tang J.-H., Yan D.-Y., Wang B.-Z., Xie Z.-J., Wu Z.-Y., Yang L. Vitamin K2 Can Rescue the Dexamethasone-Induced Downregulation of Osteoblast Autophagy and Mitophagy Thereby Restoring Osteoblast Function In Vitro and In Vivo　Frontiers in Pharmacology 2020; 11:1209
15. Yamada Y, Tada M, Mandai K. et al. Glucocorticoid use is an independent risk factor for developing sarcopenia in patients with rheumatoid arthritis: from the CHIKARA study. Clin Rheumatol 2020;39:1757–64.
16. Institute of Medicine. Dietary reference intakes for energy, carbohydrate, fiber, fat, fatty acids, cholesterol, protein, and amino acids. Washington, DC: National Academies Press, 2005. <https://www.nap.edu/catalog/10490> (24 March 2022, date last accessed).
17. EFSA Panel on Dietetic Products, Nutrition and Allergies (NDA). Scientific opinion on dietary reference values for protein. EFSA J 2012;10:2557.
18. Deutz NEP, Bauer JM, Barazzoni R. et al. Protein intake and exercise for optimal muscle function with aging: recommendations from the ESPEN Expert Group. Clin Nutr 2014;33:929–36.
19. Arends J, Bachmann P, Baracos V. et al. ESPEN guidelines on nutrition in cancer patients. Clin Nutr 2017;36:11–48.
20. Ikizler TA, Burrowes JD, Byham-Gray LD. et al. KDOQI clinical practice guideline for nutrition in CKD: 2020 update. Am J Kidney Dis 2020;76:S1–107.
21. Papageorgiou M, Dolan E, Elliott-Sale KJ, Sale C.. Reduced energy availability: implications for bone health in physically active populations. Eur J Nutr 2018;57:847–59.
22. Papageorgiou M, Martin D, Colgan H. et al. Bone metabolic responses to low energy availability achieved by diet or exercise in active eumenorrheic women. Bone 2018;114:181–8.
23. Seimon RV, Wild-Taylor AL, Keating SE. et al. Effect of weight loss via severe vs moderate energy restriction on lean mass and body composition among postmenopausal women with obesity. JAMA Netw Open 2019;2:e1913733.
24. American College of Rheumatology Ad Hoc Committee on Glucocorticoid-Induced Osteoporosis: Recommendations for the prevention and treatment of glucocorticoid-induced osteoporosis: 2001 update. Arthritis Rheum. 44(7): 1496-1503, 2001.
25. Bone and Tooth Society, National Osteoporosis Society, Royal College of Physicians: Glucocorticoid-induced osteoporosis: guidelines for prevention and treatment. London: Royal College of Physicians, 2002.
26. Nawata H, Soen S, Takayanagi R, et al.: Guidelines on the management and treatment of glucocorticoid-induced osteoporosis of the Japanese Society for Bone and Mineral Research (2004). J Bone Miner Metab. 23(2): 105-109, 2005.
27. Kanis JA, on behalf of the World Health Organization Scientific Group.: Assessment of osteoporosis at the primary health care level. WHO Collaborating Centre for Metabolic Bone Diseases; University of Scheffield, 2007.
28. Lakamwasam S, Adachi JD , Agnusdei D, et al.: A framework for the development of guidelines for the management of glucocorticoid-induced osteoporosis. Osteoporos Int. 23(9): 2257-2276, 2012.
29. Compston J, Bowring C, Cooper A, et al.: Diagnosis and management of osteoporosis in postmenopausal women and older men in the UK: National Osteoporosis Guideline Group (NOGG) update 2013. Maturitas. 75(4): 392-396, 2013.
30. Suzuki Y, Nawata H, Soen S, et al.: Guidelines on the management and treatment of glucocorticoid-induced osteoporosis of the Japanese Society for Bone and Mineral Research: 2014 update. J Bone Miner Metab. 32(4): 337-350, 2014.
31. Buckley L, Guyatt G, Fink HA, et al.: 2017 American College of Rheumatology Guideline for the prevention and treatment of glucocorticoid-induced osteoporosis. Arthritis Rheumatol. 69(8): 1521-1537, 2017.
32. Buckley L, Guyatt G, Fink HA, et al.: 2017 American College of Rheumatology Guideline for the prevention and treatment of glucocorticoid-induced osteoporosis. Arthritis Care Res. 69(8): 1095-1110, 2017.
33. Compston J, Cooper A, Cooper C, et al.: UK clinical guideline for the prevention and treatment of osteoporosis. Arch Osteoporos. 12(1): 43, 2017.
34. Gregson CL, Armstrong DJ, Bowden J, et al.: UK clinical guideline for the prevention and treatment of osteoporosis. Arch Osteoporos. 17(1): 58, 2022.
35. Lee T-H, Song Y-J, Kim H, et al.: Intervention thresholds for treatment in patients with glucocorticoid-induced osteoporosis: systematic review of guidelines. J Bone Metab. 27(4): 247-259, 2020.
36. Herath M, Langdahl B, Ebeling PR, et al.: Challenges in the diagnosis and management of glucocorticoid-induced osteoporosis in younger and older adults. Clin Endocrinol. 96(4): 460-474, 2022.
37. Kirigaya D, Nakayama T, Ishizaki T, et al.: Management and treatment of osteoporosis receiving long-term glucocorticoid treatment: current status of adherence to clinical guidelines and related factors. Intern Med. 50(22): 2793-2800, 2011.
38. Soen S, Kaku M, Okubo N, et al.: Epidemiology of glucocorticoid-induced osteoporosis and management of associate fracture risk in Japan. J Bone Miner Metab. 39(6): 1019-1030, 2021.
39. Chotiyarnwong P, McCloskey EV. Pathogenesis of glucocorticoid-induced osteoporosis and options for treatment. Nat Rev Endocrinol. 2020;16(8):437-47.
40. Buckley L, Humphrey MB. Glucocorticoid-Induced Osteoporosis. N Engl J Med. 2018;379(26):2547-56.
41. Buckley L, Guyatt G, Fink HA, Cannon M, Grossman J, Hansen KE, et al. 2017 American College of Rheumatology Guideline for the Prevention and Treatment of Glucocorticoid-Induced Osteoporosis. Arthritis Rheumatol. 2017;69(8):1521-37.
42. Leipe J, Holle JU, Weseloh C, Pfeil A, Krüger K. German Society of Rheumatology recommendations for management of glucocorticoid-induced osteoporosis. Z Rheumatol. 2021;80(Suppl 2):49-63.
43. Suzuki Y, Nawata H, Soen S, Fujiwara S, Nakayama H, Tanaka I, et al. Guidelines on the management and treatment of glucocorticoid-induced osteoporosis of the Japanese Society for Bone and Mineral Research: 2014 update. J Bone Miner Metab. 2014;32(4):337-50.
44. Ringe JD. Treatment of glucocorticoid-induced osteoporosis with calcium, vitamin D and D-metabolites. Front Horm Res. 2002;30:127-35.
45. Pereira RMR, Perez MO, Paula AP, Moreira C, Castro CHM, Zerbini CAF, et al. Guidelines for the prevention and treatment of glucocorticoid-induced osteoporosis: an update of Brazilian Society of Rheumatology (2020). Arch Osteoporos. 2021;16(1):49.
46. Ringe JD. Plain vitamin D or active vitamin D in the treatment of osteoporosis: where do we stand today? Arch Osteoporos. 2020;15(1):182.
47. Deng J, Silver Z, Huang E, Zheng E, Kavanagh K, Wen A, et al. Pharmacological prevention of fractures in patients undergoing glucocorticoid therapies: a systematic review and network meta-analysis. Rheumatology (Oxford). 2021;60(2):649-57.
48. Liu Z, Zhang M, Shen Z, Ke J, Zhang D, Yin F. Efficacy and safety of 18 anti-osteoporotic drugs in the treatment of patients with osteoporosis caused by glucocorticoid: A network
49. de Nijs RN, Jacobs JW, Algra A, Lems WF, Bijlsma JW. Prevention and treatment of glucocorticoid-induced osteoporosis with active vitamin D3 analogues: a review with meta-analysis of randomized controlled trials including organ transplantation studies. Osteoporos Int. 2004;15(8):589-602.
50. Amin S, Lavalley MP, Simms RW, Felson DT. The comparative efficacy of drug therapies used for the management of corticosteroid-induced osteoporosis: a meta-regression. J Bone Miner Res. 2002;17(8):1512-26.
51. Matsumoto T, Yamamoto K, Takeuchi T, Tanaka Y, Tanaka S, Nakano T, et al. Eldecalcitol is superior to alfacalcidol in maintaining bone mineral density in glucocorticoid-induced osteoporosis patients (e-GLORIA). J Bone Miner Metab. 2020;38(4):522-32.
52. Ringe JD, Dorst A, Faber H, Schacht E, Rahlfs VW. Superiority of alfacalcidol over plain vitamin D in the treatment of glucocorticoid-induced osteoporosis. Rheumatol Int. 2004;24(2):63-70.
53. Sambrook PN, Kotowicz M, Nash P, Styles CB, Naganathan V, Henderson-Briffa KN, et al. Prevention and treatment of glucocorticoid-induced osteoporosis: a comparison of calcitriol, vitamin D plus calcium, and alendronate plus calcium. J Bone Miner Res. 2003;18(5):919-24.
54. Chen Y, Wan JX, Jiang DW, Fu BB, Cui J, Li GF, et al. Efficacy of calcitriol in treating glucocorticoidinduced osteoporosis in patients with nephrotic syndrome: an open-label, randomized controlled study. Clin Nephrol. 2015;84(5):262-9.
55. Ringe JD, Cöster A, Meng T, Schacht E, Umbach R. Treatment of glucocorticoid-induced osteoporosis with alfacalcidol/calcium versus vitamin D/calcium. Calcif Tissue Int. 1999;65(4):337-40.
56. Ding L, Hu J, Wang D, et al. Efficacy and safety of first- and second-line drugs to prevent glucocorticoid-induced fractures. J Clin Endocrinol Metab 2020;105(1):dgz023
57. Rooney M, Bishop N, Davidson J, et al. The prevention and treatment of glucocorticoid-induced osteopaenia in juvenile rheumatic disease: a randomised double-blind controlled trial. EClinicalMedicine 2019;12:79-87
58. Soen S, Yamamoto K, Takeuchi T, et al. Minodronate combined with alfacalcidol versus alfacalcidol alone for glucocorticoid-induced osteoporosis: a multicenter, randomized, comparative study. J Bone Miner Metab 2020;38(4):511-21
59. Deng J, Silver Z, Huang E, et al. Pharmacological prevention of fractures in patients undergoing glucocorticoid therapies: a systematic review and network meta-analysis. Rheumatology (Oxford) 2021;60(2):649-57
60. Liu Z, Zhang M, Shen Z, et al. Efficacy and safety of 18 anti-osteoporotic drugs in the treatment of patients with osteoporosis caused by glucocorticoid: a network meta-analysis of randomized controlled trials. PLoS One 2020;15(12):e0243851
61. Hakala M, Kroger H, Valleala H, et al. Once-monthly oral ibandronate provides significant improvement in bone mineral density in postmenopausal women treated with glucocorticoids for inflammatory rheumatic diseases: a 12-month, randomized, double-blind, placebo-controlled trial. Scand J Rheumatol 2012;41(4):260-6
62. Reid DM, Devogelaer JP, Saag K, et al. Zoledronic acid and risedronate in the prevention and treatment of glucocorticoid-induced osteoporosis (HORIZON): a multicentre, double-blind, double-dummy, randomised controlled trial. Lancet 2009;373(9671):1253-63
63. Ward LM, Choudhury A, Also N, et al; Zoledronic acid vs placebo in pediatric glucocorticoid-induced osteoporosis: a randomized, double-blind, phase 3 trial. J Clin Endocrinol Metab 2021;106(12):e5222-35
64. Riggs BL, Hartmann LC. Selective estrogen-receptor modulators—mechanisms of action and application to clinical practice. N Engl J Med 2003; 348: 618-29.
65. Komm BS, Kharode YP, Bodine PV, et al. Bazedoxifene acetate: a selective estrogen receptor modulator with improved selectivity. Endocrinology 2005; 146:3999-4008
66. Liu Z., Zhang M., Shen Z., Ke J., et al. Efficacy and safety of 18 anti-osteoporotic drugs in the treatment of patients with osteoporosis caused by glucocorticoid: A network meta-analysis of randomized controlled trials. PLoS ONE 2020; 15:e0243851-
67. Amiche MA, Albaum JM, Tadrous M et al. Efficacy of osteoporosis pharmacotherapies in preventing fracture among oral glucocorticoid users: a network meta-analysis. Osteoporos Int. 2016 Jun;27(6):1989-98. doi: 10.1007/s00198-015-3476-4. Epub 2016 Jan 18.
68. Mok C.C., Ying K.Y. et al. Raloxifene for prevention of glucocorticoid-induced bone loss: A 12-month randomised double-blinded placebo-controlled trial. Annals of the Rheumatic Diseases 2011; 70:778-784
69. Mok C.C., Ying S.K.Y. et al. Effect of raloxifene on disease activity and vascular biomarkers in patients with systemic lupus erythematosus: Subgroup analysis of a double-blind randomized controlled trial. Lupus 2013; 22:1470-1478
70. Cho S.-K., Kim H. et al. Effectiveness of bazedoxifene in preventing glucocorticoid-induced bone loss in rheumatoid arthritis patients. Arthritis Research and Therapy 2021; 23:176
71. Buckley L., Guyatt G., Fink H.A., et al. 2017 American College of Rheumatology Guideline for the Prevention and Treatment of Glucocorticoid-Induced Osteoporosis. Arthritis Care and Research 2017; 69:1095-1110
72. Saag, K.G., E. Shane, S. Boonen, et al., Teriparatide or alendronate in glucocorticoid-induced osteoporosis, N Engl J Med, 2007. 357(20): 2028-39.
73. Saag, K.G., J.R. Zanchetta, J.P. Devogelaer, et al., Effects of teriparatide versus alendronate for treating glucocorticoid-induced osteoporosis: thirty-six-month results of a randomized, double-blind, controlled trial, Arthritis Rheum, 2009. 60(11): 3346-55.
74. Langdahl, B.L., F. Marin, E. Shane, et al., Teriparatide versus alendronate for treating glucocorticoid-induced osteoporosis: an analysis by gender and menopausal status, Osteoporos Int, 2009. 20(12): 2095-104.
75. Devogelaer, J.P., R.A. Adler, C. Recknor, et al., Baseline glucocorticoid dose and bone mineral density response with teriparatide or alendronate therapy in patients with glucocorticoid-induced osteoporosis, J Rheumatol, 2010. 37(1): 141-8.
76. Payer, J., S. Tomkova, Z. Killinger, et al., Eighteen months of teriparatide treatment leads to improvement of bone mineral density and trabecular bone score in patients with glucocorticoids induced osteoporosis: the results from prospective follow-up (registry OSTEO.sk), Clin Osteol, 2018. 23(4): 138–145.
77. Glüer, C.C., F. Marin, J.D. Ringe, et al., Comparative effects of teriparatide and risedronate in glucocorticoid-induced osteoporosis in men: 18-month results of the EuroGIOPs trial, J Bone Miner Res, 2013. 28(6): 1355-68.
78. Liu, C.L., H.C. Lee, C.C. Chen, et al., Head-to-head comparisons of bisphosphonates and teriparatide in osteoporosis: a meta-analysis, Clin Invest Med, 2017. 40(3): E146-E157.
79. Deng, J., Z. Silver, E. Huang, et al., Pharmacological prevention of fractures in patients undergoing glucocorticoid therapies: a systematic review and network meta-analysis, Rheumatology (Oxford), 2021. 60(2): 649-657.
80. Ding, L., J. Hu, D. Wang, et al., Efficacy and Safety of First- and Second-Line Drugs to Prevent Glucocorticoid-Induced Fractures, J Clin Endocrinol Metab, 2020. 105(1).
81. Tanaka, I., Y. Tanaka, S. Soen, et al., Efficacy of once-weekly teriparatide in patients with glucocorticoid-induced osteoporosis: the TOWER-GO study, J Bone Miner Metab, 2021. 39(3): 446-455.
82. Tanaka, I., Y. Tanaka, S. Soen, et al., Efficacy of once-weekly teriparatide for primary prevention of glucocorticoid-induced osteoporosis: A post hoc analysis of the TOWER-GO study, Mod Rheumatol, 2022. 32(3): 634-640.
83. Iseri K, Iyoda M, Watanabe M, et al. The effects of denosumab and alendronate on glucocorticoid-induced osteoporosis in patients with glomerular disease: A randomized, controlled trial. PLoS One. 2018 Mar;13(3):e0193846.
84. Saag KG, Wagman RB, Geusens P, et al. Denosumab versus risedronate in glucocorticoid-induced osteoporosis: a multicentre, randomised, double-blind, active-controlled, double-dummy, non-inferiority study. Lancet Diabetes Endocrinol. 2018 Jun;6(6):445-454.
85. Saag KG, Pannacciulli N, Geusens P, et al. Denosumab Versus Risedronate in Glucocorticoid-Induced Osteoporosis: Final Results of a Twenty-Four-Month Randomized, Double-Blind, Double-Dummy Trial. Arthritis Rheumatol. 2019 Jul;71(7):1174-1184.
86. Deng J, Silver Z, Huang E, et al. Pharmacological prevention of fractures in patients undergoing glucocorticoid therapies: a systematic review and network meta-analysis. Rheumatology (Oxford). 2021 Feb;60(2):649-657.
87. Liu Z, Zhang M, Shen Z et al. Efficacy and safety of 18 anti-osteoporotic drugs in the treatment of patients with osteoporosis caused by glucocorticoid: A network meta-analysis of randomized controlled trials. PLoS One. 2020 Dec;15(12):e0243851.
88. Sato AY, Cregor M, Delgado-Calle J, et al. Protection From Glucocorticoid-Induced Osteoporosis by Anti-Catabolic Signaling in the Absence of Sost/Sclerostin. J Bone Miner Res 2016; 31:1791-1802
89. Yao W, Dai W, Jiang L, et al. Sclerostin-antibody treatment of glucocorticoid-induced osteoporosis maintained bone mass and strength. Osteoporos Int. 2016; 27:283-294
90. Achiou Z, Toumi H, Touvier J, et al. Sclerostin antibody and interval treadmill training effects in a rodent model of glucocorticoid-induced osteopenia. Bone 2015; 81:691-701
91. Taylor AD, Saag KG. Anabolics in the management of glucocorticoid induced osteoporosis: an evidence-based review of long-term safety, efficacy and place in therapy. Core Evid. 2019 Aug 23;14:41-50.
92. Amiche MA, Albaum JM, Tadrous M, et al. Efficacy of osteoporosis pharmacotherapies in preventing fracture among oral glucocorticoid users: a network meta-analysis. Osteoporos Int. 2016 Jun;27(6):1989-98
93. Ding L, Hu J, Wang D, et al. Efficacy and Safety of First- and Second-Line Drugs to Prevent Glucocorticoid-Induced Fractures. J Clin Endocrinol Metab. 2020 Jan;105(1):dgz023.
94. Liu Z, Zhang M, Shen Z, et al. Efficacy and safety of 18 anti-osteoporotic drugs in the treatment of patients with osteoporosis caused by glucocorticoid: A network meta-analysis of randomized controlled trials. PLoS One. 2020 Dec;15(12):e0243851
95. Deng J, Silver Z, Huang E, et al. Pharmacological prevention of fractures in patients undergoing glucocorticoid therapies: a systematic review and network meta-analysis. Rheumatology (Oxford). 2021 Feb;60(2):649-657.
96. Ward LM, Choudhury A, Alos N, et al, Zoledoronic acid vs placebo in pediatric glucocorticoid-induced osteoporosis: A randomized, double-blind, phase3 trial. J Clin Endocrinol Metab, 2021 Nov;106(12):e5222-e5235
97. Rooney M, Bishop N, Davidson J, et al. The prevention and treatment of glucocorticoid-induced osteopaenia in juvenile rheumatic disease: A randomized double-blind controlled trial. EClinicalMedicine 2019 Jul;12:79-87
98. Noguera A, Ros JB, Pavia C, et al. Bisphosphonates, a new treatment for glucocorticoid-induced osteoporosis in children. J Ped Endocrinol Metab 2003 Apr-May;16(4):529-536
99. Nasomyont N, Hornung LN, Gordon CM, et al. Outcomes following intravenous bisphosphonate infusion in pediatric patients; A 70year retrospective chart review Bone 2019 Apr;121:60-67
100. Lim A, Simm PJ, James S, et al. Outcomes of zoledronic acid use in paediatric conditions. Horm Res Paediatr 2020;93(7-)):442-452
101. Brown JJ, Zacharin MR. Attempted randomized controlled trial of pamidronate versus calcium and calcitriol supplements for management of steroid-induced osteoporosis in children and adolescents. J Pediatr Child Health 2005 Nov;41(11):580-582
102. Inoue Y, Mitsunaga K, Yamamoto T, et al. Early use of alendronate as a protective factor against the development of glucocorticoid-induced bone loss in childhood-onset rheumatic diseases: a cross-sectional study. Pediatr Rheumatol Online 2018 Jun;16(1):36
103. Inoue Y, Shimojo N, Suzuki S, et al. Efficacy of intravenous alendronate for the treatment of glucocorticoid-induced osteoporosis in children with autoimmune diseases. Clin Rheumatol 2008 Jul;27(7):909-912
104. Suzuki Y, Nawata H, Soen S, et al.: Guidelines on the management and treatment of glucocorticoid-induced osteoporosis of the Japanese Society for Bone and Mineral Research: 2014 update. J Bone Miner Metab. 32(4): 337-350, 2014.
105. Yoshimura N, Muraki S, Oka H, et al. ：Prevalence of knee osteoarthritis, lumbar spondylosis, and osteoporosis in Japanese men and women: the research on osteoarthritis/osteoporosis against disability study. J Bone Miner Metab.27:620–628, 2009.
106. Soen S, Kaku M, Okubo N, et al.: Fracture risk associated with glucocorticoid‑induced osteoporosis in Japan. Journal of Bone and Mineral Metabolism. published online 11May 2022
107. Gregson CL, Armstrong DJ, Bowden J, et al.: UK clinical guideline for the prevention and treatment of osteoporosis. Arch Osteoporos. 17(1): 58, 2022.
108. Lakamwasam S, Adachi JD, Agnusdei D, et al.: A framework for the development of guidelines for the management of glucocorticoid-induced osteoporosis. Osteoporos Int. 23(9): 2257-2276, 2012.
109. EULAR evidence‐based recommendations on the management of systemic glucocorticoid therapy in rheumatic diseases 2007
110. Herath M, Langdahl B, Ebeling PR, et al.: Challenges in the diagnosis and management of glucocorticoid-induced osteoporosis in younger and older adults. Clin Endocrinol. 96(4): 460-474, 2022.
111. Leipe J, Holle J, Weseloh C, et al.: German Society of Rheumatology recommendations for management of glucocorticoid-induced osteoporosis. Z Rheumatol publish online.27: October 2021
112. Sato A, Richardson D, Cregor M,　et al.: Glucocorticoids induce bone and muscle atrophy by tissue-specific mechanisms upstream of E3 ubiquitin ligases. Endocrinology. 158(3):664–677,2017.
113. Orimo H, Yaegashi Y, Onoda T, et al.: Hip fracture incidence in Japan: estimates of new patients in 2007 and 20-year trend. Arch Osteoporosis 4:71-77, 2009.
114. Tamaki J, et al.: Total 25-hudroxyvitamin D levels predict fracture risk: results from the 15-year follow-up of the Japanese Population-based Osteoporosis (JPOS) Cohort Study. Osteoporosis Int 28:1903-1913, 2017.
115. Ruggiero S, Dodson T, Fantasia J, et al.: American Association of Oral and Maxillofacial Surgeons Position Paper on Medication-Related Osteonecrosis of the Jaw—2014 Update. J Oral Maxillofac Surg 72:1938-1956, 2014.
116. Black D, Geiger E, Eastell R, et al.: Atypical Femur Fracture Risk versus Fragility Fracture Prevention with Bisphosphonates. N Engl J Med. 383:743-53, 2020.
117. Saag KG, Emkey R, Schnitzer TJ, et al. Alendronate for the prevention and treatment of glucocorticoid-induced osteoporosis. Glucocorticoid-Induced Osteoporosis Intervention Study Group. N Engl J Med. 1998; 339(5): 292-9.
118. Cohen S, Levy RM, Keller M, et al. Risedronate therapy prevents corticosteroid-induced bone loss: a twelve-month, multicenter, randomized, double-blind, placebo-controlled, parallel-group study. Arthritis Rheum. 1999; 42(11): 2309-18.
119. Roux C, Reid DM, Devogelaer JP, et al. Post hoc analysis of a single IV infusion of zoledronic acid versus daily oral risedronate on lumbar spine bone mineral density in different subgroups with glucocorticoid-induced osteoporosis. Osteoporos Int. 2012; 23(3): 1083-90.
120. Buckley L, Guyatt G, Fink HA, et al. 2017 American College of Rheumatology Guideline for the Prevention and Treatment of Glucocorticoid-Induced Osteoporosis. Arthritis Rheumatol. 2017; 69(8): 1521-37.
121. Langdahl BL, Marin F, Shane E, et al. Teriparatide versus alendronate for treating glucocorticoid-induced osteoporosis: an analysis by gender and menopausal status. Osteoporos Int. 2009; 20(12): 2095-104.
122. Boonen S, Wahl DA, Nauroy L, Brandi ML, Bouxsein ML, Goldhahn J, Lewiecki EM, Lyritis GP, Marsh D, Obrant K, Silverman S, Siris E, Akesson K. Balloon kyphoplasty and vertebroplasty in the management of vertebral compression fractures. Osteoporos Int 2011;22: 2915-34.
123. Schmidt AH, Asnis SE, Haidukewych G, Koval KJ, Thorngren KG. Femoral neck fractures. Instr Course Lect 2005;54: 417-45.
124. Meyer C, Chang J, Stern P, Osterman AL, Abzug JM. Complications of distal radial and scaphoid fracture treatment. J Bone Joint Surg Am 2013;95: 1517-26.
